# Multiomics Blood-Based Biomarkers Predict Alzheimer’s Predementia with High Specificity in a Multicentric Cohort Study

**DOI:** 10.14283/jpad.2024.34

**Published:** 2024-02-06

**Authors:** B. Souchet, A. Michaïl, M. Heuillet, A. Dupuy-Gayral, E. Haudebourg, C. Pech, A. Berthemy, F. Autelitano, B. Billoir, K. Domoto-Reilly, C. Fowler, T. Grabowski, S. Jayadev, C. L. Masters, Jérôme Braudeau

**Affiliations:** 1 AgenT, 4 rue Pierre Fontaine, 91000 Evry-Courcouronnes, France; 2 EVOTEC, 195 Route d’Espagne, 31100 Toulouse, France; 3 Department of Neurology, University of Washington, 1410 NE Campus Pkwy, Seattle, WA 98195 USA; 4 Department of Molecular Imaging & Therapy, Austin Health, Austin Hospital, Heidelberg, VIC 3084 Australia; 5 The Florey Institute of Neuroscience and Mental Health, The University of Melbourne, Parkville, VIC 3052 Australia

**Keywords:** Blood-based biomarkers, Alzheimer’s predementia, animal model, machine learning, mass spectrometry

## Abstract

**Background:**

The primary criteria for diagnosing mild cognitive impairment (MCI) due to Alzheimer’s Disease (AD) or probable mild AD dementia rely partly on cognitive assessments and the presence of amyloid plaques. Although these criteria exhibit high sensitivity in predicting AD among cognitively impaired patients, their specificity remains limited. Notably, up to 25% of non-demented patients with amyloid plaques may be misdiagnosed with MCI due to AD, when in fact they suffer from a different brain disorder. The introduction of anti-amyloid antibodies complicates this scenario. Physicians must prioritize which amyloid-positive MCI patients receive these treatments, as not all are suitable candidates. Specifically those with non-AD amyloid pathologies are not primary targets for amyloid-modifying therapies. Consequently there is an escalating medical necessity for highly specific blood biomarkers that can accurately detect pre-dementia AD, thus optimizing amyloid antibody prescription.

**Objectives:**

The objective of this study was to evaluate a predictive model based on peripheral biomarkers to identify MCI and mild dementia patients who will develop AD dementia symptoms in cognitively impaired population with high specificity.

**Design:**

Peripheral biomarkers were identified in a gene transfer-based animal model of AD and then validated during a retrospective multi-center clinical study.

**Setting:**

Participants from 7 retrospective cohorts (US, EU and Australia).

**Participants:**

This study followed 345 cognitively impaired individuals over up to 13 years, including 193 with MCI and 152 with mild dementia, starting from their initial visits. The final diagnoses, established during their last assessments, classified 249 participants as AD patients and 96 as having non-AD brain disorders, based on the specific diagnostic criteria for each disorder subtype. Amyloid status, assessed at baseline, was available for 82.9% of the participants, with 61.9% testing positive for amyloid. Both amyloid-positive and negative individuals were represented in each clinical group. Some of the AD patients had co-morbidities such as metabolic disorders, chronic diseases, or cardiovascular pathologies.

**Measurements:**

We developed targeted mass spectrometry assays for 81 blood-based biomarkers, encompassing 45 proteins and 36 metabolites previously identified in AAV-AD rats.

**Methods:**

We analyzed blood samples from study participants for the 81 biomarkers. The B-HEALED test, a machine learning-based diagnostic tool, was developed to differentiate AD patients, including 123 with Prodromal AD and 126 with mild AD dementia, from 96 individuals with non-AD brain disorders. The model was trained using 70% of the data, selecting relevant biomarkers, calibrating the algorithm, and establishing cutoff values. The remaining 30% served as an external test dataset for blind validation of the predictive accuracy.

**Results:**

Integrating a combination of 19 blood biomarkers and participant age, the B-HEALED model successfully distinguished participants that will develop AD dementia symptoms (82 with Prodromal AD and 83 with AD dementia) from non-AD subjects (71 individuals) with a specificity of 93.0% and sensitivity of 65.4% (AUROC=81.9%, p<0.001) during internal validation. When the amyloid status (derived from CSF or PET scans) and the B-HEALED model were applied in association, with individuals being categorized as AD if they tested positive in both tests, we achieved 100% specificity and 52.8% sensitivity. This performance was consistent in blind external validation, underscoring the model’s reliability on independent datasets.

**Conclusions:**

The B-HEALED test, utilizing multiomics blood-based biomarkers, demonstrates high predictive specificity in identifying AD patients within the cognitively impaired population, minimizing false positives. When used alongside amyloid screening, it effectively identifies a nearly pure prodromal AD cohort. These results bear significant implications for refining clinical trial inclusion criteria, facilitating drug development and validation, and accurately identifying patients who will benefit the most from disease-modifying AD treatments.

**Electronic Supplementary Material:**

Supplementary material is available in the online version of this article at 10.14283/jpad.2024.34.

## Introduction

Alzheimer’s Disease (AD) diagnosis is based on a cognitive impairment evaluation alongside a brain amyloid deposits estimation through measurements of Aβ42/40 peptides and/or p-tau protein in cerebrospinal fluid (CSF) or by positron emission tomography (PET) imaging (1). These criteria introduce the biological diagnostic framework encompassing probable AD dementia (2) and Mild Cognitive Impairment (MCI) due to AD (3). However, amyloid deposition is common in cognitively impaired patients suffering from a brain disorder excluding AD (Non-AD BD) (4-7).

When used to identify patients that will develop AD dementia symptoms within the MCI population, tests relying on amyloid deposition exhibit high sensitivity (67–86%) but low specificity (47–72%), resulting in false positive rate of up to 30%: these individuals identified as AD actually have a non-AD BD (4–7) (Supplementary fig.l). Blood tests currently under development have shown promising results for the non-invasive brain amyloid deposits prediction (8). The plasmatic Aβ42/40 ratio and p-tau assays have achieved areas under the receiver operating characteristic curve (AUROC) of up to 87% and up to 95% respectively in predicting brain amyloid plaques (9, 10). These assays correlate strongly with PET imaging and CSF measurements. However, they share similar specificity limitations in predicting the development of AD dementia symptoms. Deep investigations into plasma p-tau 217 demonstrated its efficacy in differentiating amyloid-positive AD dementia patients from amyloid-negative non-AD BD patients, with a sensitivity of 93% and specificity of 89% (11). But this assay’s specificity drops to 47% in distinguishing amyloid-positive AD dementia patients from amyloid-positive non-AD BD patients (11). Recently, plasma MTBR-Tau243 has been identified as a potential indicator of both amyloid and Tau PET status, along with MMSE scores at the time of blood draw (12). Nonetheless, the capability of MTBR-Tau243 to predict the onset of AD dementia symptoms remains unexplored. The concurrent prediction of amyloid plaques and tangles by plasma tau biomarkers suggests that these two mechanisms are not independent, and that once amyloidosis is established, tangle formation appears to be systematic independently of the patient pathology. Notably, the co-presence of amyloid and tau biomarkers ((A+T+) CSF profile) is prevalent in non-AD brain disorders. For instance, 29% of Parkinson’s Disease (PD) patients and 40% of those with Lewy Body Dementia (LBD) exhibit this CSF profile (13). Among LBD patients, 21% test tau-positive on PET scans, with 75% also showing amyloid positivity (14). Moreover, amyloid and tau brain lesions are commonly found in elderly individuals without cognitive impairment (15). These observations suggest that blood-based tau biomarkers, such as plasma p-tau 217 and MTBR-Tau243, are more indicative of amyloid and tau status than being distinct predictors of AD dementia symptoms.

Anti-amyloid treatments, such as lecanemab and donanemab, are coming to market and will soon have to be prescribed by neurologists. However, current projections show that treating all patients who meet the FDA-recommended criteria, those exhibiting mild cognitive impairment or mild dementia alongside amyloid positivity, will not be feasible due to complex logistics and high treatment costs (16). Projected utilization of Lecanemab (Leqembi®) is estimated at 2.5 million patients by 2030 (https://www.reuters.com/business/healthcare-pharmaceuticals/ us-fda-approveseisai-biogens-alzheimers-drug-2023-01-06/), a figure roughly equivalent to the number of AD cases in France (17). This necessitates a strategic approach by clinicians in identifying patients who are most likely to benefit from this therapy. The challenge lies in discerning the optimal candidates for treatment, particularly in light of the significant side effects associated with anti-amyloid antibodies. Clinicians might be inclined to delay prescription until overt symptoms of cognitive decline specific to AD are evident, using these symptoms as a trigger for initiating treatment. However, this cautious approach carries the risk of patients’ conditions advancing beyond the stage where they would be eligible for effective treatment.

Current data reveals that 29% of non-AD BD patients exhibit amyloid positivity (5). These amyloid-positive MCI patients, who will develop non-AD BD symptoms not attributable to amyloid accumulation (18-24), do not appear to be prime candidates for amyloid-modifying therapy. Consequently, there is an urgent need to develop novel blood biomarkers capable of rapidly and accurately identifying amyloid-positive patients at high risk of developing AD dementia symptoms. This specific subpopulation should be the focus for prioritizing anti-amyloid treatments, considering the optimal balance between clinical efficacy and potential side effects.

We report a novel discovery method leveraging the AAV-AD rat, a gene transfer-based animal model (25), to pre-identify blood AD pre-dementia biomarkers. Our approach unveiled alterations in peripheral blood metabolism that accurately segregate patients that will develop AD dementia symptoms among cognitively impaired individuals. The developed B-HEALED™ test demonstrates a specificity of 93% in this regard. Notably, the combination of a positive amyloid status and a positive B-HEALED test result yields a remarkable 100% specificity in predicting the onset of AD dementia symptoms among the cognitively impaired patients. Interestingly, these peripheral metabolism changes involve biological pathways similar to those described at the cerebral level in AD. Our findings indicate the potential of peripheral metabolism markers to predict AD symptom onset with high specificity, up to 13 years prior to the manifestation of dementia. These insights have profound implications for enhancing clinical trial participant selection, streamlining drug development and validation, and effectively identifying patients most likely to benefit from disease-modifying AD therapies (26).

## Methods

### AA V-AD rat model induction

The animal model was induced in 8-week-old male rats through a gene transfer approach following a methodology previously described (25). All experiments were conducted in accordance with the ethical standards of French and European regulations (European Communities Council Directive 2010/63/EU, authorization number APAFIS#4449-2016031012491697).

### Rat plasma collection

Plasma samples from the groups of animals (25, 27, 28), were collected, quickly frozen and stored at −80°C until mass spectrometry (MS) analysis. Specifically, whole blood was obtained through intra-cardiac puncture from anesthetized, non-fasting rats, using K2 EDTA-coated tubes. These tubes were then maintained on ice for 30 minutes to 3 hours before undergoing centrifugation at 1500g for 10 minutes at 4°C. Subsequently, the plasma supernatant was divided into three aliquots for distinct MS analyses: proteomic, metabolomic, and lipidomic. Each aliquot was promptly frozen in liquid nitrogen and preserved at −80°C. The duration between plasma collection and MS analysis ranged from 1 to 5 years.

### Untargeted mass spectrometry methods

We conducted untargeted proteomic profiling of plasma samples from both rats (Discovery study) and humans (Transferability study) using Hyper Reaction Monitoring (HRM™) SpeeD Mass Spectrometry (Biognosys AG, Switzerland) (29). Additionally, untargeted metabolomic analysis was performed using LC-MS/MS and Polar LC platforms, supported by proprietary software (Metabolon, Inc., USA) (30).

### Biomarker discovery in AAV-AD rats

The raw data for each feature underwent a normalization process. This involved adjusting with reference samples, applying a base-2 logarithmic transformation, and standardizing by subtracting the mean and dividing by the standard deviation, thereby bringing all features onto a common scale for subsequent analysis. Any constituent not quantified across all rats or exhibiting negligible variance was removed from the analysis to ensure only significant and informative features were included.

Recursive Feature Elimination (RFE) with cross-validation was carried out to further refine our feature set, employing a variety of algorithms including random forest, gradient tree boosting, lasso, elastic net, perceptron, linear kernel support vector machine, and logistic regression. These algorithms ranked the blood components based on their significance, facilitating the systematic removal of the least informative ones.

After reducing the number of features, sequential feature selection (either backward or forward) with cross-validation was employed. During this process, the most informative feature was added or removed at each step, based on the cross-validation score of the algorithm under consideration. Several algorithms were used for this purpose, including logistic regression, linear SVM, Gaussian kernel SVM, random forest, a perceptron with two small hidden layers, and nearest neighbor classifier.

This process culminated in the identification of a subset of biomarkers indicative of AD. Notably, when a minimum of three blood biomarkers belonged to the same compound family, that entire family was classified as an AD biomarker set.

### Diagnostic criteria used to label participants

To evaluate the efficacy of novel AD blood biomarkers, the diagnostic criteria and assessment protocols employed to label the participants should be consistent with those used for determining the diagnostic performance of biomarkers related to amyloid deposition (5-7, 31). The cognitive status of participants, categorized as either MCI or dementia, was defined at the time of blood collection based on recognized criteria. Participant labels (AD or Non-AD BD) were established based on the complete clinical follow-up: the clinical diagnosis was made at the advanced stages of each pathology using established reference standards, such as the NINCDS-ADRDA criteria or the Diagnostic and Statistical Manual of Mental Disorders, Fourth Edition (DSM-IV). Therefore, the retrospective labeling at the time of blood sampling considered the patient’s cognitive decline during the clinical follow-up based on the reference standard (Supplementary Fig.2). Informed consent for the use of biological samples and associated clinical data in research was obtained from all participants, as documented by each clinical partner.

### Patients plasma collection

Whole blood samples were obtained via intravenous puncture, collected in EDTA K2-coated tubes. The extraction protocol varied across sampling centers, with specific details outlined in Supplementary Table 1. Following collection, the plasma supernatant was promptly allocated into aliquots designated for subsequent analyses. These aliquots were immediately frozen and preserved at ™80°C until MS analysis was conducted. The interval between plasma collection and the initiation of MS analysis spanned from 1 to 25 years.

### Linear discriminant analysis

Linear discriminant analyses were performed using the ‘svd’ solver, which corresponds to a singular value decomposition, within the scikit-learn package. Prior to analysis, all datasets were normalized by subtracting the mean and dividing by the standard deviation, ensuring standardized data for effective comparison. The results of the linear discriminant analysis were then visualized by projecting the studied classes onto two axes, utilizing the Seaborn Python package for graphical representation.

### Plasma constituent informativeness

Each biomarker was independently normalized for each cohort by subtracting the median value of healthy control individuals (HC). Normalized MS values were then merged and further normalized through subtraction of the biomarker mean and division by the standard deviation. A random selection process was employed, selecting n biomarkers (where n ranged from 1 to 50) from the detected 128 biomarkers. The efficacy of these biomarkers in identifying AD patients was assessed through 5-fold cross-validation using logistic regression, limited to these n biomarkers. This procedure was repeated 500 times for each n value, facilitating the evaluation of AD detection accuracy from the prodromal phase with each randomly selected biomarker set. The same methodology was applied to the remaining blood constituents, enabling comparison of performance between the pre-identified rat biomarkers and other blood constituents. All analyses were conducted using the scikit-learn Python package (https://scikit-learn.org/stable/).

### Targeted mass spectrometry methods

From the 128 biomarkers investigated during the transferability study, 103 were selected based on the technical feasibility of developing targeted mass spectrometry (MS) methods. High-throughput liquid chromatography-tandem mass spectrometry (LC-MS/MS) quantification methods were developed for 50 proteins and 53 metabolites.

For proteomics, a robust and repeatable absolute quantification workflow was set up. The sample preparation method was developed using the Bravo automated liquid handling platform (Agilent Technologies, Inc., USA) and the PreOmics sample preparation kit (PreOmics GmbH, Germany), starting with 2 *μ*L of plasma. Each digested plasma sample was analyzed in a targeted mode (Multiple Reaction Monitoring, MRM) using a triple quadrupole instrument coupled with high-performance liquid chromatography (HPLC). Optimization of parameters such as collision energies and gradient length allowed for the selection of optimal proteotypic peptides and their transitions, leading to the quantification of 70 peptides for 45 proteins. Limits of detection and quantification were determined for each protein.

For metabolomics, two different LC-MS/MS methods were developed using a single preparation method based on liquid-liquid extraction, starting with 100 *μ*L of plasma. For each method, the HPLC and MS parameters were optimized. For each metabolite, specific Selected Reaction Monitoring (SRM) transitions were determined, and the absolute quantification was calculated based on calibration curves performed in solvent. Limits of detection and quantification were determined for each metabolite. Metabolites that were not detected (10), under the limit of quantification or not reproducible (7) were excluded, resulting in a final list of 36 quantified metabolites.

The 372 human plasma samples from 7 independent neurological cohorts were randomized in 9 analytical batches and quantified independently. To simulate routine clinical practice, the batches were analyzed sequentially with at least a week’s gap between each. The assays, conducted between December 2021 and May 2022, were blinded, with MS researchers unaware of sample details. Different quality controls (QCs) were performed to ensure good reproducibility of sample preparation across the batches and reliability of the LC-MS measurements. Proteomic data integration was performed using Skyline software (MacCoss Lab, University of Washington in Seattle, USA), with manual peak area review and absolute quantification via an in-house script. Metabolomic data treatment utilized TraceFinder software (Thermo Fisher Scientific, Inc., USA), with each sample manually reviewed and metabolites quantified against a calibration curve for each standard.

### Machine learning procedures

Predictive machine learning (ML) models were developed by selecting the most relevant biomarkers from the 81 measured. The models were trained on 49% of the samples (training dataset) and internally validated on a separate 21% (validation dataset). During this phase, the positivity cutoff and various parameters were optimized. After finalizing and locking the ML model, its performance was assessed through internal validation.

In AD biomarker validation, blind external validation is frequently omitted, which can make the interpretation of the results challenging due to limited confidence in their generalizability and a high risk of overfitting (32). To avoid overfitting, the locked predictive ML model was then tested on an independent test dataset (external validation) (32, 33), to ensure the results reliability and reproducibility. To mitigate experimental bias, the samples were randomized across the three datasets based on 6 criteria: amyloid status (positive, negative or not determined), APOE genotype, analytical batch, clinical cohort (7 independent cohorts), gender (male or female) and clinical label (healthy controls, asymptomatic AD, non-AD BD, prodromal AD, AD dementia).

Machine learning models were trained and evaluated on normalized data sets by subtracting the mean and dividing by the standard deviation. The best algorithm typology (e.g., logistic regression, SVM, random forest, neural network) to be used was evaluated by comparing the performances of ranked biomarker sets defined by the mRMR feature selection method (34). Once the best algorithm typology had been defined (linear algorithms) on the training set, we determined the best biomarkers set based on an improved method of Sequential Backward Floating Selection, still on this same set by cross-validation, then by blind validation on the independent validation set.

Once the optimal algorithm typology and the best predictive biomarkers were defined, the selection of the best hyperparameters of the linear algorithm was performed both by grid search with cross-validations on the training set and in such a way that the performances were maintained in validation on the validation set. All the cross-validations performed were successions of 10× 10-fold stratified cross-validations, and successions of 100× 10-folds cross-validations during the final performance evaluation phases. As the algorithm returns a probability, we defined the threshold above which an individual is predicted to develop AD dementia symptoms. Finally, once the set of algorithm hyperparameters and the biomarkers were determined, the algorithm was trained on merged training and validation sets before being evaluated in blind conditions on the test set. The test set was also normalized in blind conditions, by subtracting the mean and dividing the standard deviation of merged training and validation sets. The performance metrics used were balanced accuracy, sensitivity, and specificity. The specificity was operationalized as the proportion of patients who, during clinical follow-up, were diagnosed with clinical symptoms associated with brain disorders other than Alzheimer’s Disease (AD), as per established reference standards like the NINCDS-ADRDA criteria or the Diagnostic and Statistical Manual of Mental Disorders, Fourth Edition (DSM-IV) and exhibiting a predictive model score below the established positivity threshold of 0.76. Conversely, sensitivity was defined as the proportion of patients identified during clinical follow-up as exhibiting clinical AD dementia symptoms, according to the same reference standards, and who register a score exceeding the positivity threshold of 0.76 in the predictive model (35). All methods were implemented by using Python packages scikit-learn (https://scikit-learn.org/stable/), mRMR (https://github.com/smazzanti/mrmr) and mlxtend (http://rasbt.github.io/mlxtend/).

### Comparative analysis with amyloid tests

We performed a comparative analysis including participants with amyloid status available at baseline. We calculated the predictive performance (specificity, sensitivity, and false positive rate) based on amyloid status, the B-HEALED predictive model, and a combination of both. In the combined approach, participants testing positive in both the amyloid test and the B-HEALED model were classified as positive.

### Statistical analysis

Data are expressed as the mean + standard deviation (SD). Statistical, including receiver operating characteristic (ROC) curves, were performed using GraphPad Prism 9.5 software (GraphPad Software, LLC, USA). The statistical significance was set to a p-value < 0.05 for all tests. One-way ANOVA followed by Holm-Šídák’s multiple comparisons post hoc test or 2-way ANOVA were used to determine the significance of differences between groups. Student’s t-test was used when only 2 groups were analyzed. Pearson correlation test was used to determine linear correlation between two variables. Chi square test or McNemar’s chi-square test was used for comparison of two distributions. 2-way ANOVA was used to determine the significance of differences between groups with one main effect (one source of variation). 3-way ANOVA was used to determine the significance of differences between groups for two main effects (two sources of variation).

## Results

### Pre-identification of blood biomarkers in AAVAD rats

**Figure 1:**
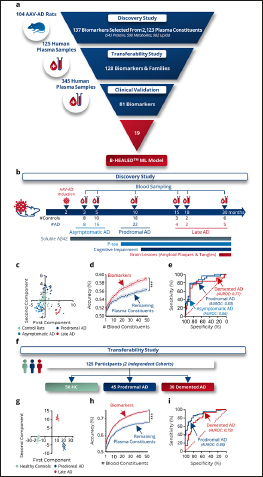
Identification and validation of blood biomarkers for Alzheimer’s disease a, Development steps for predictive machine learning models. The development was subdivided into 3 steps: Biomarker discovery in 104 AAV-AD rats, Transferability study on 125 human plasma samples, and Clinical validation study on 345 human plasma samples, b–e, Biomarker discovery step, b, Plasma from 47 controls and 57 AAV-AD rats was collected throughout the life of the animal at the asymptomatic, prodromal and demented stages. Plasma analysis enabled the identification of 137 biomarkers or families of biomarkers among all the blood constituents measured that are informative about AD status in rats, c, Linear discriminant analysis on all determined biomarkers confirms a different distribution of AAV-AD rats from control rats, d, Comparison of informativeness of identified biomarkers to the rest of the plasma constitution. Two-way ANOVA. ***p<0.0001. e, ROC curves based on the predictions of 100× 10-fold cross-validations with all biomarkers. f–i, Transferability clinical study, f, Plasma from 50 cognitively healthy individuals and 75 AD patients was collected at the prodromal (n=45) and demented (n=30) stages. Of the 137 biomarkers or biomarker families, 128 could be measured, g, Linear discriminant analysis on these 128 pre-identified biomarkers confirms a different distribution of AD patients from healthy controls, h, Comparison of informativeness of identified biomarkers to the rest of the plasma constitution. Two-way AN OVA. ***p<0.0001. i, ROC curves based on the predictions of 100× 10-fold cross-validations with all biomarkers.

We analyzed the blood profile of 104 AAV-AD and control rats across various stages of AD, ranging from asymptomatic to advanced AD stages (25, 27, 28). Samples were analyzed by untargeted mass spectrometry to measure the relative concentration of 2,123 plasma constituents, encompassing 543 proteins, 598 metabolites, and 982 lipids (Fig.1a). Machine learning (ML) approaches identified 137 biomarkers or families of biomarkers informative about the rats’ AD status (Fig.1b). Subsequent linear discriminant analysis (LDA) of these biomarkers revealed a clear spatial segregation between control and AD rats (Fig.1c). When these biomarkers were combined, their predictive capacity to differentiate AAV-AD rats from controls was significantly enhanced compared to other plasma constituents (p<0.0001; Fig.1d), yielding AUROCs ranging from 77.7% to 85.7%, depending on the stage of AD progression (Fig.1e).

### AAV-AD rats biomarkers are informative in humans

To confirm the applicability of the biomarkers pre-identified in the AAV-AD rats to humans, 125 plasma samples collected from cognitively normal, prodromal AD or demented AD participants were analyzed by untargeted mass spectrometry (Fig.1f, Supplementary Fig.3a). Among the 137 biomarkers pre-identified in rats, 128 were detected in human plasma and measured. Linear discriminant analysis (LDA) of these 128 biomarkers demonstrated distinct spatial segregation among healthy controls (HC), prodromal AD, and AD demented participants (Fig.1g, Supplementary fig.3b–c). The combined diagnostic power of these biomarkers was significantly greater than that of other plasma constituents (p<0.0001; Fig.1h), yielding AUROCs of 79.2% for prodromal AD and 88.6% for AD demented patients (Fig.1i). These results confirmed the relevance of the identified blood biomarkers for predicting AD in human subjects.

### Multiplexed MS targeted assays are reproducible

Recognizing the limitations of untargeted mass spectrometry (MS) assays in routine clinical practice, we developed targeted MS assays for 81 biomarkers identified during the transferability study. The reproducibility of these methods was evaluated by calculating coefficients of variation (%CV), which ranged from 0.03% to 38.30%, with an average of 9.9% + 0.8 (Supplementary 4a). The validity of plasma concentration measurements was assessed by comparing the concentrations measured in the samples from cognitively unimpaired participants (n= 239) with the concentrations of 68 biomarkers referenced in the literature. This comparison revealed a significant correlation (p<0.0001; r^2^=0.78; Supplementary Fig.4b), confirming the efficacy of our multiplexed MS assays compared to each existing single method. Additionally, the assays were validated for linearity, sensitivity, specificity, precision, and repeatability, ensuring their robustness for clinical application.

### Participants reflective of real-world population

**Figure 2:**
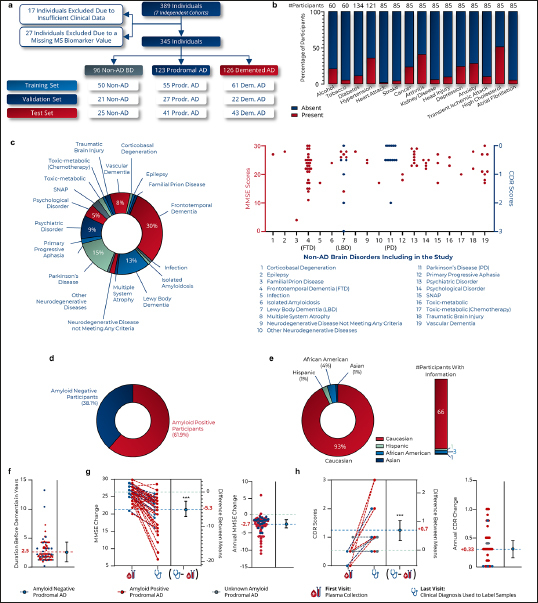
Characteristics of clinical study participants a, Distribution of samples according to the training, validation and test data sets, b, Prevalence of comorbidities in participants for whom information was available, c, Distribution of 19 pathologies among non-AD BD participants (left panel). MMSE (red dots) and CDR (blue dots) scores of Non-AD BD patients at the time of blood collection (right panel). FTD: Frontotemporal Dementia, LBD: Lewy Body Dementia, PD: Parkinson Disease, d, Percentage of amyloid positive participants in the validation study, e, Distribution of ethnic background among participants for whom information was available. The study is predominantly composed of Caucasian participants (93%), which may introduce bias in interpreting results for non-Caucasian populations. Further studies are required to validate these findings in cohorts with participants of Hispanic, African American, and Asian ethnicities, f, Duration from blood draw to diagnosis of AD dementia in prodromal AD participants, g, Change in MMSE scores during the clinical follow-up and associated annual change, h, Change in CDR scores during the clinical follow-up and associated annual change. Student’s T test. ***p<0.001.

Our study analyzed a large and diverse dataset of 389 plasma samples (each sample represents an individual participant) (Fig.Fig.2a). These samples were drawn from seven distinct retrospective neurological cohorts, encompassing a broad range of nationalities and geographic locations (Supplementary Tables 2–9).

Among all participants, 17 were excluded due to insufficient clinical data. The 372 remaining plasma samples were then randomized according to 6 parameters, including the mass spectrometry batch (Supplementary Fig.5). The plasma samples were analyzed over 9 MS analytical batches. 27 participants (6.9%) were subsequently excluded from the analysis due to the absence of at least one MS biomarker value. Ultimately, 345 plasma samples were included in the study (Fig.2a).

To mirror real-world conditions, patients with various comorbidities (Fig.2b) and a spectrum of non-AD BD pathologies (Fig.2c) were included. Among the participants, 82.9% had known amyloid status at blood sampling, with 61.9% testing amyloid positive (Fig.2d, Supplementary Table 2, Supplementary Fig.6). This distribution aimed to reflect the general population’s amyloid positivity rates (36), albeit with slight deviations: amyloid-negative cognitively impaired AD patients were over-represented (24.2%) compared to the 15% described in the literature (37), and amyloid-positive non-AD BD patients were under-represented (18.9%) relative to the 29% reported in literature (5).

Supplementary tables provide a detailed description of each cohort (Supplementary tables 3–9). A notable limitation was the disproportionate representation of Caucasian participants (93%) compared to African American (4%), Hispanic (1%), and Asian (1%) origins (Fig.2e).

For prodromal AD participants, the average duration from the blood sample collection to the AD dementia symptoms onset was 2.5+1.9 years (Fig.2f). Amyloid-positive prodromal AD patients converted more quickly to AD dementia (2.3+1.4 years) than amyloid-negative prodromal AD patients (3.3+3.1 years, p=0.04). On average, prodromal AD patients experienced a decline of 5.3 Mini-Mental State Examination (38) (MMSE) points (−2.7 MMSE points/year) and an increase of 0.7 Clinical Dementia Rating (39) (CDR) points (+0.33 CDR points/year) (Fig.2g–h).

### B-HEALED test predicted AD with 93.0% specificity

**Figure 3:**
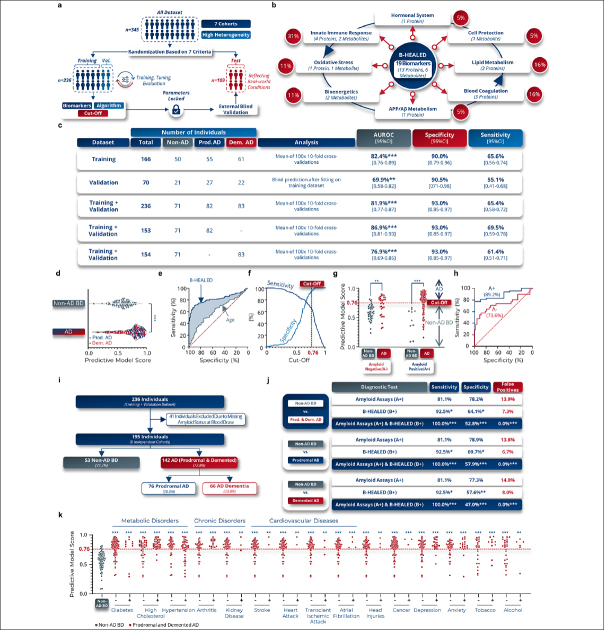
B-HEALED test predicts Alzheimer’s disease patients among cognitively impaired individuals a, Machine learning predictive models development validated with internal and external blind validations, b, Biological pathways in which the 19 selected peripheral biomarkers are involved. The red lines represent the links between Alzheimer’s disease and each of the biological pathways identified, while the blue lines represent the interactions between the different biological pathways, c, Performances obtained with a cut-off value of 0.76 by the ML model trained on blood concentrations of 19 biomarkers and 1 covariate (age at blood sampling) in terms of AUROC, specificity and sensitivity on the training, validation and training-validation datasets. Means and 95% confidence intervals. *p<0.05, **p<0.01, ***p<0.001. d-f, Performances obtained with a cut-off value of 0.76 by the ML model trained on blood concentrations of 19 biomarkers and 1 covariate (age at sampling) in terms of AUROC, specificity and sensitivity. Means and 95% confidence intervals during the internal validation, e-i, Internal validation performances, d, Scores predicted by the ML model as a function of the clinical label in 100× 10-fold cross-validations on training +validation dataset (internal validation). One-way ANOVA, ***p<0.001. e, ROC curve of 100× 10-fold cross-validations, f, Evolution of sensitivity and specificity according to the cut-off used by the ML model to predict clinical status, g, Predictive model scores based on tested subject amyloid status and clinical label. 2-way ANOVA followed by Tukey’s post hoc test. ***p<0.001. h, ROC curves according to the amyloid status of the tested subject, i-j, Comparative analysis with amyloid status during internal validation, i, Participants included in the comparative analysis with amyloid, j, Performances obtained during the comparative analysis. False positive rates were calculated considering a 60% Alzheimer’s prevalence among cognitively impaired individuals. Chi-square test compared to Non-AD BD values as reference, *p<0.05, **p<0.01, ***p<0.001. k, Scores predicted by the ML model as a function of the comorbidities in AD patients. One-way ANOVA followed by Holm-Šídák’s multiple comparisons post hoc test with the non-AD BD clinical group as the reference. **p<0.01, ***p<0.001.

After analyzing the training dataset (Supplementary table 10), we selected 19 blood biomarkers (13 proteins and 6 metabolites) and one covariate (age at blood draw) as the most informative panel to differentiate AD from non-AD BD patients (Fig.3b). These biomarkers, which are produced or regulated by peripheral organs, play roles in AD-associated biological pathways such as innate immune response (40) (31%), blood coagulation (41) (16%), lipid metabolism (42) (16%), bioenergetics (43) (11%), oxidative stress (44) (11%), APP/Aβ metabolism (45) (5%), cell protection (46) (5%), and sex hormonal system (47) (5%) (Fig.3b). Algorithms were then trained to achieve specificities above 85% and validated on the test dataset to identify the most effective, robust, and reproducible predictive algorithm (Fig.3c).

During internal validation (training + validation datasets), the predictive ML model was calibrated to a cut-off of 0.76, resulting in 93.0% specificity in predicting AD patients (prodromal AD (n=82) and AD dementia (n=83)) from non-AD BD patients (n=71), with 65.4% sensitivity (AUROC=81.9%, p<0.0001) (Fig.3c–f). The comparative analysis of patients with prodromal AD and those with AD dementia revealed a similar predictive accuracy between the two groups. Specifically, the sensitivity was observed to be 69.5% in prodromal AD patients and 61.4% in AD dementia patients (Fig.2c). With all parameters and hyper-parameters locked in, we established the B-HEALED predictive model (incorporating the biomarkers panel, the hyperparameters, and trained ML algorithm). Quality controls confirmed the B-HEALED test’s performance consistency across all 7 cohorts studied (Supplementary Fig.7a), as well as in 19 different of brain disorders (Supplementary fig.7b). The test’s performance was not affected by covariates such as age, gender, or APOE e4 genotype (Supplementary fig.7c–f). The B-HEALED test strongly outperformed MMSE or CDR score in predicting AD (Supplementary fig.7g).

Our study revealed that the B-HEALED test successfully predicted AD during internal validation for both amyloid-positive (AUROC=89.2%, p<0.0001) and amyloid-negative (AUROC=73.6%, p<0.0001) participants (Fig.3g–h). These findings suggest that the B-HEALED test and amyloid deposit-related tests provide different yet complementary information. To explore the relationship between amyloid status and the B-HEALED test, we conducted a comparative analysis involving 195 participants with available amyloid status at blood collection (Fig.3i). In this analysis, 62.1% of participants were positive for amyloid deposit-related tests (A+), 48.7% for the B-HEALED test (B+), and 38.5% for both tests (A+/B+). The amyloid deposit-related tests and the B-HEALED predictive model did not identify the same participants (McNemar’s chi-square test: p=0.002). When we calculated the performance of amyloid deposit-related tests to predict AD patients from non-AD BD participants, we observed 81.1% specificity and 78.2% sensitivity, resulting in a 13.9% false positive rate considering a 60% AD prevalence among cognitively impaired individuals (Fig.3j). This calculated false positive rate is lower than the literature-reported rate, which can be explained by an underrepresentation of amyloid-positive non-AD BD patients (18.9% of amyloid-positive non-AD BD patients) in our study compared to the 29% cited in the literature (5). Nevertheless, these results align with performances described in the literature, underscoring the limitations of tests based solely on cerebral amyloid deposit estimates for identifying AD patients with high specificity (5-7).

In contrast, the B-HEALED test predicted AD from non-AD BD patients with 92.5% specificity and 64.1% sensitivity, resulting in a 7.3% false-positive rate (Fig.3j). This head-to-head comparison highlights the B-HEALED test’s superior specificity relative to amyloid deposit-based tests (p=0.02): B-HEALED biomarkers reduced the false positive rate by nearly 50% compared with tests based on amyloid status. When participants positive for both (A+/B+) were considered AD, we reached 100% specificity of and 52.8% sensitivity, resulting in 0% false positives (Fig.3j). This comparative analysis demonstrates the advantage of combining the two tests to minimize the false positive rate, as opposed to using the amyloid deposit-related tests alone (chi-square test: p<0.0001). The analysis demonstrated that, upon integration with amyloid status, the sensitivity and false-positive rates yielded comparable results for both prodromal AD and AD dementia patients when assessed independently. Notably, a slightly elevated sensitivity was observed in the prodromal AD group, registering at 57.9%, in contrast to 47.0% observed in the AD dementia group (Fig.3j).

### B-HEALED test was not impacted by comorbidities

To assess whether comorbidities influence the B-HEALED test’s predictive scores, we conducted analyses comparing scores between non-AD BD patients and AD patients with known comorbidity status. We examined 15 different comorbidities, including metabolic disorders, chronic disorders and cardiovascular pathologies. The results of this analysis indicated that these comorbidities did not significantly affect the B-HEALED test’s predictive scores (Fig.3k).

### B-HEALED test proves robustness in blind external validation

**Figure 4:**
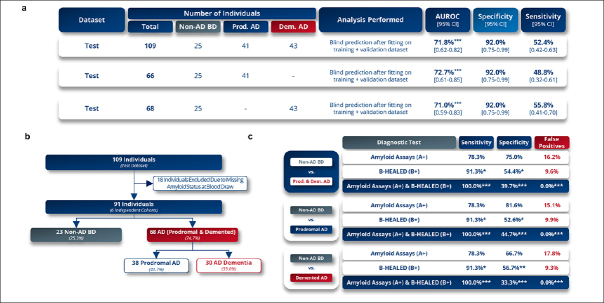
B-HEALED test robustly predicts Alzheimer’s disease patients among cognitively impaired individuals a, Performances obtained during the external blind validation (test set) in terms of AUROC, specificity and sensitivity. Means and 95% confidence intervals with a cut-off value of 0.76. b–c, Comparative analysis with amyloid status during external validation, b, Participants included in the comparative analysis with amyloid, c, Performances obtained during the comparative analysis. False positive rate was calculated considering a 60% Alzheimer’s prevalence among cognitively impaired individuals. Chi-square test compared to amyloid tests values as reference, *p<0.05, **p<0.01, ***p<0.001.

In the external validation phase, the B-HEALED test achieved 92.0% specificity in predicting AD, including prodromal AD (n=41) and AD dementia (n=43), from non-AD BD patients (n=25), with 52.4% sensitivity (AUROC=71.8%, p=0.001) (Fig.4a). The comparative analysis of patients with prodromal AD and those with AD dementia revealed a similar predictive accuracy between the two groups. Specifically, the sensitivity was observed to be 48.8% in prodromal AD patients and 55.8% in AD dementia patients (Fig.4a). These findings strongly argue against model overfitting and affirm the robustness of the B-HEALED predictive model. A head-to-head comparison confirms that the B-HEALED test outperforms the specificity of tests based on cerebral amyloid deposits estimation (p=0.01) in the external validation. When participants positive for both (A+/B+) were considered as having AD, 100% specificity and 39.7% sensitivity were achieved, resulting in 0% false positives (Fig.4b–c). This combination led to a significant reduction in false positive rate compared to the amyloid deposit-related tests used alone (16.2% false positive rate, considering a 60% Alzheimer’s prevalence among cognitively impaired individuals; chi-square test: p<0.0001). The analysis demonstrated that, upon integration with amyloid status, the sensitivity and false-positive rates yielded comparable results for both prodromal AD and AD dementia patients when assessed independently. Notably, a slightly elevated sensitivity was observed in the prodromal AD group, registering at 44.7%, in contrast to 33.3% observed in the AD dementia group (Fig.4c).

## Discussion

**Figure 5:**
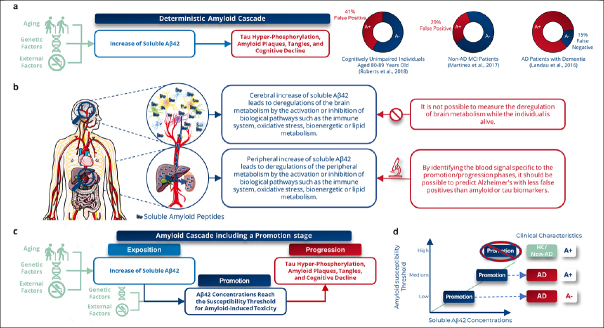
Diagnosing Alzheimer’s disease thanks to biomarkers produced by peripheral organs a, The prevailing view of Alzheimer’s disease (AD) is largely grounded in the amyloid hypothesis, which posits a deterministic cascade of events triggered by an elevation in soluble amyloid peptides within the brain, leading to the deposition of extracellular amyloid plaques, tau intra-neuronal aggregation, neurodegeneration, and ultimately, cognitive impairment. However, this model falls short in accounting for the fact that up to 41% of cognitively normal individuals with amyloid deposits and 29% of non-AD BD patients with mild cognitive impairment (MCI) show positive results on amyloid PET imaging, while 15% of demented AD patients exhibit negative amyloid PET scans. b, This study provides evidence to support the possibility of predicting AD, with a lower false-positive rate observed in both amyloid-positive and negative individuals. This detection relies on a distinct AD plasma signature derived from peripheral organs, where an increase in the concentration of soluble amyloid at the brain and peripheral level triggers similar biological pathways in these two compartments in the context of AD. Notably, this peripheral signature can predict AD from the predementia stage, despite being unmeasurable at the brain level, c, We propose a novel amyloid theory, modulated by inter-individual susceptibility, based on equivalent performances in both amyloid-positive and negative subjects. Our theory involves three key stages: exposition, promotion, and progression. During the promotion stage, an increase in soluble Ap42 peptide leads to tau hyper-phosphorylation, amyloid plaques, tangles, and eventually dementia. Brain or peripheral Ap42 concentrations will reach the susceptibility threshold for amyloid-induced toxicity during this stage, leading to pathological pathway engagement, d, This threshold is different for each individual, which could explain why up to 15% of symptomatic AD patients are amyloid-negative (low susceptibility threshold) and up to 41% of cognitively unimpaired elderly are amyloid-positive (high susceptibility threshold). Peripheral metabolism deregulations would enable to predict that the threshold is reached (promotion).

The soluble Aβ42 peptide (oligomer or protofibril forms) has been identified as the most toxic molecule responsible for AD symptoms (48). Consequently, this molecule certainly constitutes a valuable biomarker for predicting patients that will develop AD dementia symptoms. Unfortunately, no method currently exists for estimating the intracerebral Aβ42 concentration during a patient’s lifetime, and these soluble forms have only been observed in human brains through post-mortem biochemical analyses of AD symptomatic patients’ cerebrums (49). As a result, the current AD diagnosis, as defined by the NIA-AA criteria, relies on a cognitive assessment (at least MCI) and the estimation of cerebral amyloid deposits (referred to as amyloid status) through amyloid PET scans or by determining Aβ42 and/or p-tau levels in the CSF (2, 3). However, this approach presents specificity challenges: 29% of MCI patients who will develop non-AD BD symptoms are amyloid-positive (5) (Fig.5a), and up to 40% are both CSF amyloid and tau positive (13). These patients meet the NIA-AA criteria, which explains the low specificity of amyloid and tau deposit-related tests (CSF assays or PET imaging) to predict which MCI patients will progress to AD dementia, as described in Cochrane reviews (5-7, 31). Thus, brain amyloid and tau lesion load biomarkers (via imaging, CSF, or plasma) demonstrate higher sensitivity (estimated at 81.5% (35)) compared to the B-HEALED predictive model but exhibit lower specificity (estimated at 66.5% (35)). Consequently, these biomarkers are associated with a greater false-positive rate in identifying patients at risk of developing AD dementia.

A substantial body of evidence indicates that amyloid plaques are not the underlying cause of the observed symptoms in non-AD BD. For patients with conditions such as Parkinson’s disease (18), Lewy body dementia (19), cortical basal syndrome (20), post-stroke neurodegeneration (21), schizophrenia (22), alcohol-related cognitive disorders (23), and late-life depression (24), amyloid status (positive or negative) does not significantly influence symptom severity or cognitive decline rate. Therefore, amyloid-positive non-AD BD patients, despite being eligible for anti-amyloid therapies, may not experience slowed cognitive decline from these treatments. Prescribing anti-amyloid treatment for these patients seems questionable due to an unfavorable tradeoff between clinical benefits and potential side effects.

This observation highlights that amyloid-positive MCI patients that will develop non-AD BD symptoms (5) do not seem to be the primary candidates for treatment. There is a critical need to develop highly specific blood biomarkers that enable clinicians to quickly identify patients for whom the risk of AD dementia symptoms onset is very high among the amyloid-positive population. This subpopulation represents the ideal target for anti-amyloid antibody treatments due to the favorable balance between clinical benefits and side effects. With the introduction of anti-amyloid antibodies, the need for highly specific blood biomarkers thus becomes crucial to prevent over-treatment of amyloid-positive patients that will develop Non-AD BD symptoms.

The B-HEALED predictive model holds promise as a high-specificity test, offering a potential alternative for the efficient and accurate identification of patients most likely to benefit from anti-amyloid therapies. Its deployment could facilitate quicker prescriptions of anti-amyloid antibodies by better pinpointing patients with a favorable clinical benefit-to-side effect ratio. The proposed logistical process for its deployment would involve patients diagnosed with MCI visiting a medical analysis laboratory within a partner network. Their blood would be drawn, and plasma extracted with two aliquots prepared. These samples would then be sent to a central laboratory equipped for mass spectrometry to perform the B-HEALED biomarker assays. Parallel amyloid blood biomarker tests would be conducted as companion diagnostics, with the choice of test depending on the treatment’s mode of action. Currently, given that only anti-amyloid drugs have regulatory approval, the envisaged companion tests are amyloid assays such as Aβ42, Aβ40, p-Tau 181, p-tau 217, and p-tau 231, or their combinations. The data would be securely transferred to a web server compliant with health data hosting standards, where the B-HEALED model would analyze the MS data and provide a predictive score. Patients identified as positive by both the amyloid test and the B-HEALED model would be prioritized for anti-amyloid treatments, ensuring a targeted and efficient approach to therapy allocation.

Normal aging is associated with increased concentrations of soluble Aβ42 peptide in the blood (50) and brain, correlating with higher prevalence of cerebral amyloid pathology in individuals without dementia (51) and in those with non-AD BD (52). Interestingly, the amyloid-positive subjects proportion in individuals aged 70 to 79 with no cognitive impairment and those in age-matched non-AD BD patients is very similar (33% (53) and 29% (5) respectively). This similarity suggests a common mechanism leading to spontaneous amyloid positivity. This age-related amyloid deposition, however, does not clarify why many elderly individuals with positive amyloid biomarkers do not develop AD dementia symptoms. It is possible that these individuals have a greater resilience and resistance to soluble Aβ42 accumulation, amyloid plaques, and neurofibrillary tangles than patients that will develop AD symptoms (15, 54). Given these findings, focusing solely on the concentration of soluble Aβ42 in biofluids or the presence of amyloid deposition leads to a high false positive rate in predicting AD symptom onset. A more effective strategy might involve assessing the pathological consequences of these molecules. Such an approach could distinguish between patients who will develop AD symptoms and spontaneously amyloid-positive individuals who are resilient to amyloid-induced toxicity. This refined diagnostic method could substantially reduce the rate of false positives in AD prediction (Fig.5b).

To identify highly specific AD biomarker, it is imperative to obtain access to blood samples from patients collected from the prodromal phase and longitudinally followed until the onset of AD or Non-AD BD dementia symptoms. However, the slow progression of AD and the subsequent scarcity of longitudinally collected blood samples with comprehensive clinical follow-up present significant challenges. This issue is exacerbated by the ‘curse of dimensionality’—a disparity between the high number of molecular features (blood constituents) per sample, often in the thousands, and the limited number of available samples, resulting in a sample-to-feature ratio of less than one (55). The complexity of obtaining a sufficiently large and diverse human blood sample set, encompassing a range of geographic origins, comorbidities, and types of brain disorders, further hinders the robustness and generalizability of potential pre-dementia blood biomarkers. This diversity is crucial in the discovery phase to ensure confidence in the biomarkers’ applicability across various patient populations (56). Consequently, there is currently no universally accepted set of blood biomarkers for pre-dementia patients capable of predicting the onset of AD dementia symptoms, validated through machine learning (ML) techniques.

Addressing the bottleneck in biomarker discovery necessitates innovative approaches. One strategy involves initially identifying blood markers in AD animal models, followed by the application of these findings to human sample analysis. Traditional transgenic or Aβ-induced animal models, which simulate genetic forms of AD, are not ideal for discovering blood biomarkers that can specifically predict the onset of sporadic AD dementia symptoms (57). To overcome this, we utilized a gene transfer-based animal model (25) designed to mimic sporadic AD in terms of biochemistry, cognitive symptoms, histology, and aging similar to humans. In this controlled setting, we collected plasma samples at key stages of AD pathology development. Using machine learning (ML) methods, we pre-identified 137 informative AD blood biomarkers. Subsequently, 81 of these biomarkers were validated in a limited human blood sample set. This approach enabled the development of precise predictive ML models while maintaining an advantageous sample-to-feature ratio, thereby avoiding the pitfalls associated with ‘small data’ conditions (ensuring a sample/features ratio greater than 1).

The high specificity of the B-HEALED test to predict the development of AD dementia symptoms may be explained by a three-stage AD progression model, inspired by the model of carcinogenesis (58) (Fig.5c; Supplementary Fig.8; Supplementary discussion). The first stage, “exposition”, results from increased soluble Aβ42 concentration in the brain and periphery, influenced by factors such as age (59), genetic (60), or modifiable factors (61). The second stage, “promotion”, is triggered when Aβ42 concentration surpasses the individual’s unique susceptibility threshold for Aβ42 toxicity, which can vary from one individual to another. This variability could explain why some individuals develop amyloid plaques without displaying AD symptoms (high threshold) and why some AD patients develop symptoms even though they had not yet tested positive for amyloid deposits (low threshold). Finally, the “progression” phase involves the chain of events associated with AD pathology, including deregulation of metabolism, amyloid and tau deposition, neurodegeneration, and progressive cognitive AD symptoms.

In line with this mechanism, the amyloid cascade hypothesis is compatible with the existence of AD patients who are highly sensitive to soluble Aβ42 toxicity but do not yet exhibit amyloid deposition (amyloid-negative AD patients) (62). In contrast, amyloid-positive non-AD BD patients are amyloid-resilient (15) despite having sufficient intracerebral concentrations of soluble Aβ42 to result in its deposition, but insufficient concentrations to induce its neurotoxic effects leading to AD dementia symptoms. This distinction underscores the importance of detecting a blood biomarker signature indicative of the specific AD progression stage. Such detection could enable the prediction of patients who will develop AD dementia symptoms with high specificity, irrespective of their amyloid status at the time of testing. It would also aid in avoiding misdiagnosis of amyloid-positive non-AD BD patients by considering the individual’s resilience or resistance to amyloid pathology. Identifying these stage-specific signatures in blood could be a pivotal step towards more accurate and personalized AD diagnosis.

Here we demonstrate that peripheral multiomics plasma signatures, reflecting the downstream consequences of Aβ42 oligomer-induced toxicity, can predict the development of AD dementia symptoms with a higher level of specificity than current amyloid tests. This represents a significant advancement in the development of novel blood-based diagnostic tools. Specifically, the B-HEALED model represents an innovative blood test generation that can be used alone or in combination with existing tests. It shows particular efficacy in identifying a predominantly MCI patient population that will progress to AD dementia symptoms, making it a valuable tool for clinical trial recruitment. Additionally, the B-HEALED test can assist in the selection of MCI patients for anti-amyloid drug prescriptions, enhancing the benefit-to-risk ratio by significantly reducing false positives compared to tests solely based on amyloid deposition. To further validate and integrate this model into clinical practice, efforts are being made to conduct larger-scale clinical trials involving diverse cohorts. Simultaneously, we are working to ensure that assay methods are compliant with Clinical Laboratory Improvement Amendments (CLIA), Food and Drug Administration (FDA), and European Medicines Agency (EMA) guidelines, with the aim of expediting the clinical application of these tests.

### Supplementary Materials

Multiomics blood-based biomarkers predict Alzheimer’s predementia with high specificity in a multicentric cohort study
