# Supplementary material for: Multiomics Blood-Based Biomarkers Predict Alzheimer’s Predementia with High Specificity in a Multicentric Cohort Study
Source: J Prev Alzheimers Dis. 2024 Feb 6;11(3):567–81. doi: 10.14283/jpad.2024.34 (PMC11061038; doi:10.14283/jpad.2024.34)
Supplement: Supplementary file 1 — Multiomics blood-based biomarkers predict Alzheimer’s predementia with high specificity in a multicentric cohort study [file 42414_2024_319_MOESM1_ESM.docx]

**Supplementary Materials**

**Title:**

Multiomics blood-based biomarkers predict Alzheimer’s predementia with high specificity in a multicentric cohort study

**Authors:**

Benoît Souchet, Alkéos Michaïl, Maud Heuillet, Aude Dupuy-Gayral, Eloi Haudebourg, Catherine Pech, Antoine Berthemy, François Autelitano, Baptiste Billoir, Kimiko Domoto-Reilly, Christopher Fowler, Thomas Grabowski, Suman Jayadev, Colin L. Masters, Jérôme Braudeau

Table of contents

[Supplementary Figures 3](#_Toc153880343)

[Supplementary Figure 1 3](#_Toc153880344)

[Supplementary Figure 2 4](#_Toc153880345)

[Supplementary Figure 3 5](#_Toc153880346)

[Supplementary Figure 4 6](#_Toc153880347)

[Supplementary Figure 5 7](#_Toc153880348)

[Supplementary Figure 6 8](#_Toc153880349)

[Supplementary Figure 7 9](#_Toc153880350)

[Supplementary Figure 8 10](#_Toc153880351)

[Supplementary Tables 11](#_Toc153880352)

[Supplementary Table 1 11](#_Toc153880353)

[Supplementary Table 2 12](#_Toc153880355)

[Supplementary Table 3 13](#_Toc153880356)

[Supplementary Table 4 14](#_Toc153880357)

[Supplementary Table 5 15](#_Toc153880358)

[Supplementary Table 6 16](#_Toc153880359)

[Supplementary Table 7 17](#_Toc153880360)

[Supplementary Table 8 18](#_Toc153880361)

[Supplementary Table 9 19](#_Toc153880362)

[Supplementary Table 10 20](#_Toc153880363)

[Supplementary Table 11 21](#_Toc153880364)

[Supplementary Table 12 22](#_Toc153880365)

[Supplementary discussion 24](#_Toc153880366)

[Supplementary References 26](#_Toc153880367)

# Supplementary Figures

Supplementary Figure 1


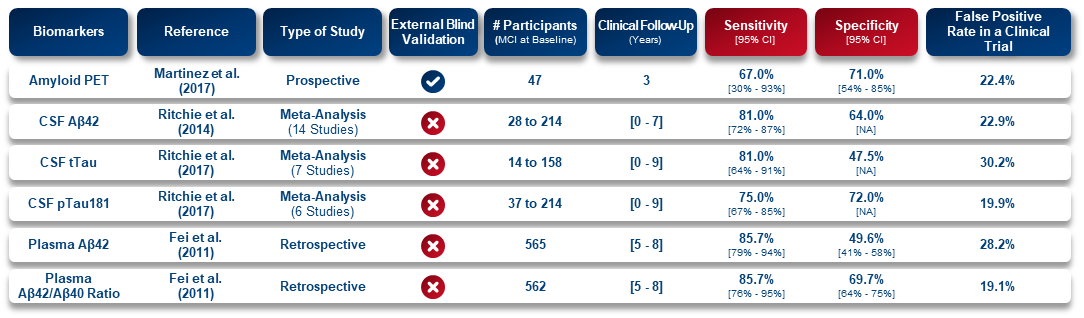


***Sup. Fig.1 | Analyses of the diagnostic performance of current biomarkers.*** *Tests to identify prodromal AD patients among those with MCI primarily rely on amyloid and/or tau biomarkers. However, the specificity of these tests ranges only from 47% to 72%, contributing to a relatively high false positive rate. Consequently, their use for participant selection in clinical trials focused on prodromal AD can result in false positive rates between 19% and 30%, assuming a 60% prevalence of Alzheimer's disease among MCI individuals. Studies lacking external blind validation often derive optimal cut-off levels from Receiver Operating Characteristic (ROC) analyses conducted on their entire sample set. Without a pre-specified cut-of these studies tend to overestimate the reported accuracy.*

Supplementary Figure 2


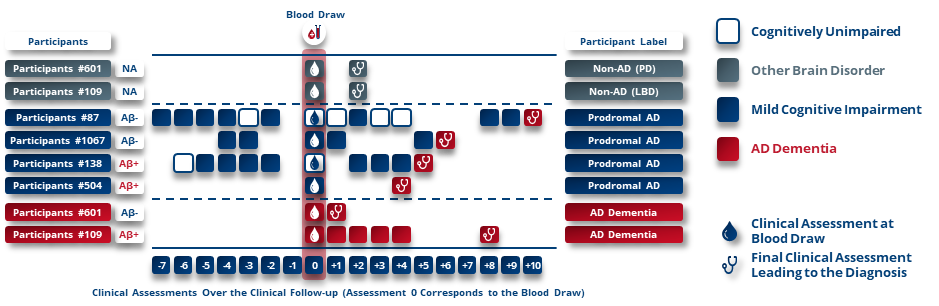


***Sup. Fig.2 | Examples of participants labels based on clinical follow-up.***

Supplementary Figure 3


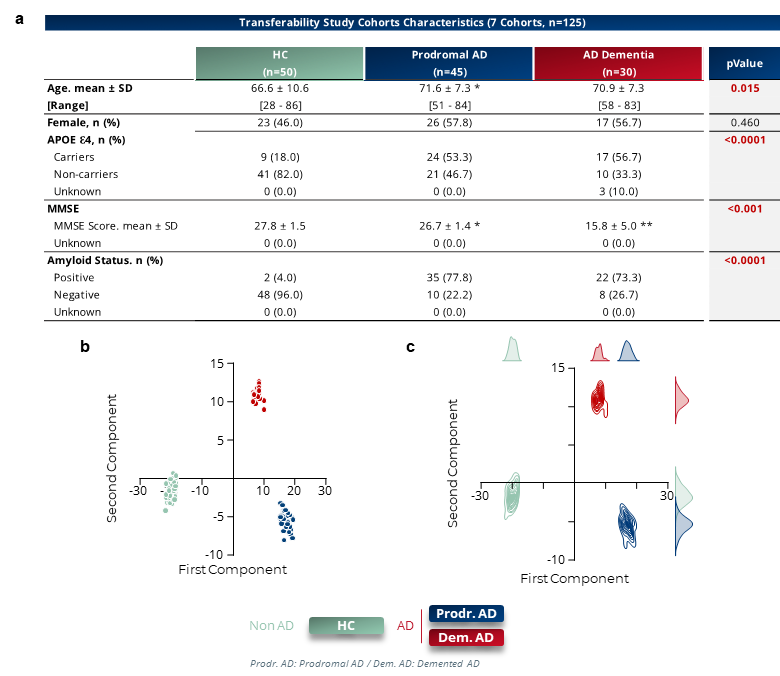


***Sup. Fig.3 | Transferability Study of blood biomarkers for Alzheimer's disease: Quality controls. a****, Clinical characteristics of the samples included in the transferability study: healthy controls (HC), prodromal AD and demented AD patients.* ***b-c,*** *LDA based on the 128 identified biomarkers in HC, prodromal AD and AD dementia patients.* ***d****, Comparison of the informative value of the 128 identified biomarkers against the remaining plasma constituents. The difference in informativeness between the pre-selected biomarkers in the rat model and the rest of the human plasmatic constitution confirms the transferability of the biomarkers identified in the AAV-AD rat to a human context. Two-way ANOVA. ***p<0.001.*

Supplementary Figure 4

*
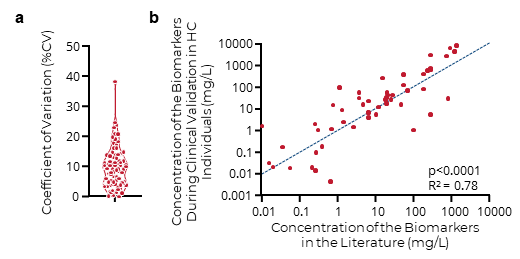
*

***Sup. Fig.4 | Targeted Mass spectrometry assays validation: Development of targeted methods for 81 biomarkers. a****, Assay methods were developed for 81 pre-identified biomarkers in the AAV-AD rat and validated in the transferability study. Coefficients of variation (%) were determined for each assay.* ***b****, Analysis of the concentrations of the 68 blood biomarkers in healthy controls by the developed targeted MS methods and those found in the literature on fresh plasma confirm the accuracy of the assays obtained by multiplexed methods compared with each of the methods described in the literature for each biomarker measured alone but also support the absence of biomarker degradation resulting from the sample storage. Pearson Correlation Test.*

Supplementary Figure 5

*
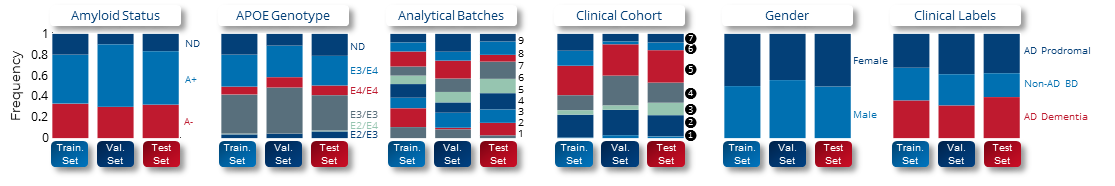
*

***Sup. Fig.5 | Sample randomization.*** *Distribution of samples in the three datasets according to amyloid status, APOE genotype, analytical batch, original cohort (❶ GeneMatch, ❷ AIBL, ❸ University of Washington Cohort, ❹ SPIN ❺ BioCogBank, ❻ Washington University Cohort, ❼ Stanford School of Medicine Cohort), gender, and clinical label used by predictive machine learning models. ND, Not Determined. Non-AD BD, Brain disorders excluding AD.*

Supplementary Figure 6

***Sup. Fig.6 | Amyloid status in studied samples.*** *Details on how amyloid status was determined for each cohort. No standardized protocol exists for determining amyloid status. Slight variations may therefore exist in the test, biomarkers, cut-offs, and kits used for each cohort. Mean ± SD. Student's t test.*

Supplementary Figure 7

***
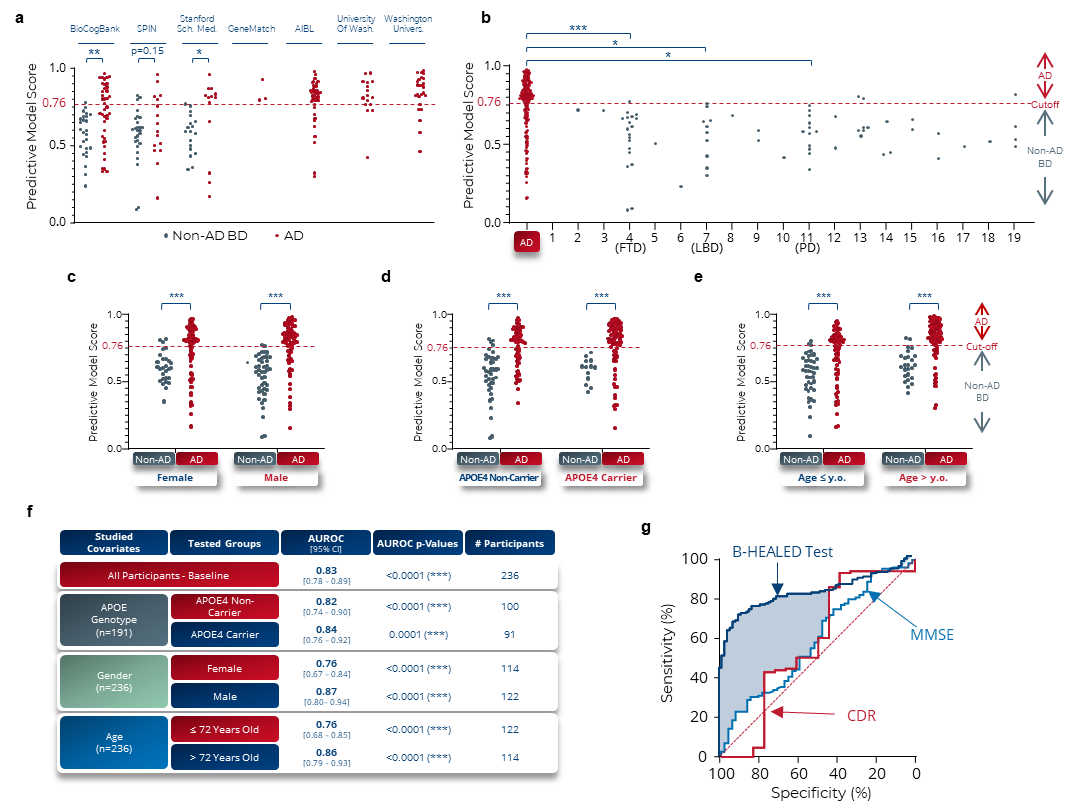
***

***Sup. Fig.7 | B-HEALED test robustly predicts AD patients among cognitively impaired individuals: Quality controls and covariates impact on predictive model performances. a****, Distribution of pathologies among Non-AD BD participants.* ***b****, MMSE and CDR scores of Non-AD BD patients at the time of blood collection. FTD: Frontotemporal Dementia, LBD: Lewi Body Dementia, PD: Parkinson Disease.* ***c****, Performances obtained with a cut-off of 0.76 by the ML model trained on blood concentrations of 19 biomarkers and 1 covariate (age at blood sampling) in terms of AUROC, specificity and sensitivity. Means and 95% confidence intervals. *p<0.05, **p<0.01, ***p<0.001.* ***d-j****, Quality controls performed on the Training-Validation dataset (Internal validation).* ***d****, Predictive scores according to each tested cohort. Student T test. *p<0.05, **p<0.01.* ***e****, Predictive scores according to each brain disorder. One-way ANOVA followed by Holm-Šídák's multiple comparisons post hoc test. *p<0.05, ***p<0.001.* ***f****, Predictive scores in function of participant’s gender. One-way ANOVA followed by Holm-Šídák's multiple comparisons post hoc test. ***p<0.001.* ***g****, Predictive scores in function of APOE4 genotype. One-way ANOVA followed by Holm-Šídák's multiple comparisons post hoc test. ***p<0.001.* ***h****, Predictive scores in function of participant’s age. One-way ANOVA followed by Holm-Šídák's multiple comparisons post hoc test. ***p<0.001.* ***i****, Summary of APOE4 genotype, gender and age impact in terms of AUROC.* ***j****, ROC curves obtained with cognitive scores (MMSE or CDR) compared with the performance of the study biomarkers.* ***k-l****, Comparative analysis with amyloid status during external validation.* ***m****, Participants included in the comparative analysis with amyloid.* ***n****, Performances obtained during the comparative analysis. False positive rate were calculated considering a 60% Alzheimer’s prevalence among cognitively impaired individuals. Chi-square test compared to amyloid tests values as reference, *p<0.05, **p<0.01, ***p<0.001.*

Supplementary Figure 8


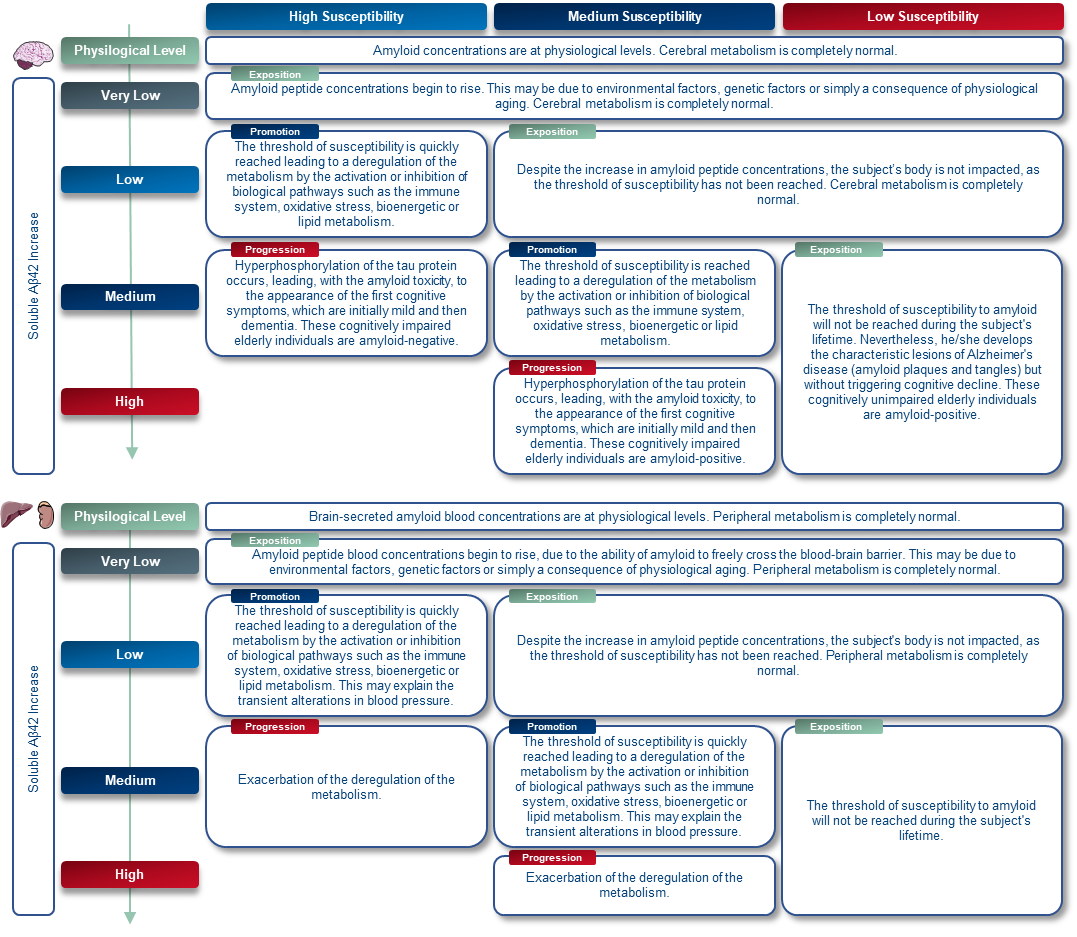


***Sup. Fig.10 | Hypothesis of brain and peripheral responses to amyloid peptide increase according to the intra-individual susceptibility threshold.***

# Supplementary Tables

Supplementary Table 1

Supplementary Table 2


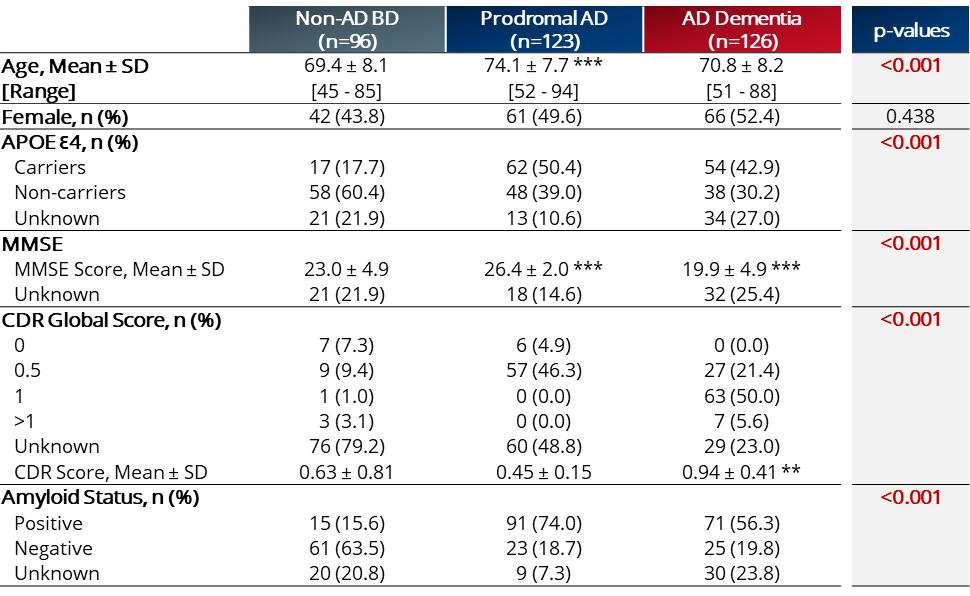


***Sup. Table 2 | Clinical validation – Cohorts characteristics.*** *Age, MMSE and CDR scores,* *one-way ANOVA followed by Holm-Šídák’s multiple comparisons post hoc test. Gender, APOE4 and amyloid status, chi-square test. Post hoc analyses were performed with the non-AD BD clinical group as the reference. *p<0.05, **p<0.01, ***p<0.001.*

Supplementary Table 3


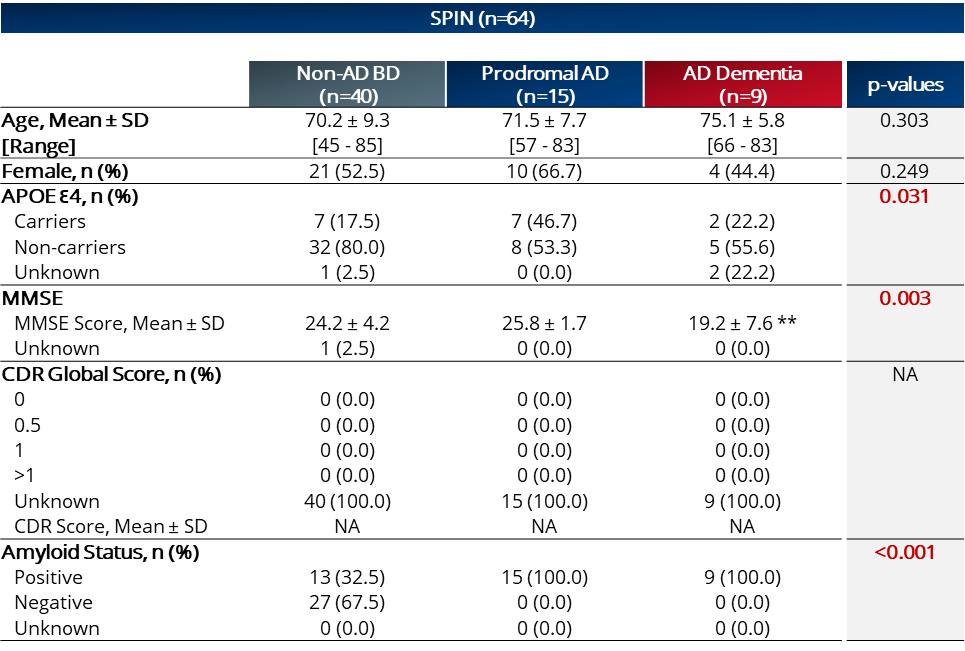


***Sup. Table 3 | SPIN (1) (Barcelona), Spain – Cohort characteristics.*** *Age, MMSE and CDR scores,* *one-way ANOVA followed by Holm-Šídák's multiple comparisons post hoc test. Gender, APOE4 and amyloid status, chi-square test. Post hoc analyses were performed with the non-AD BD clinical group as the reference. *p<0.05, **p<0.01, ***p<0.001. NA, Not Applicable.*

Supplementary Table 4

***
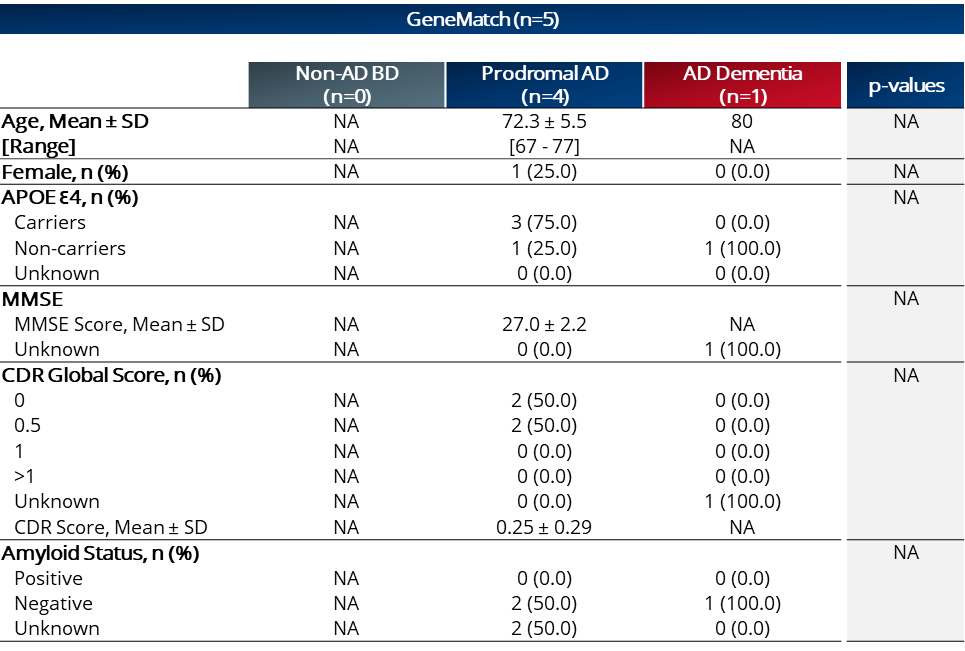
***

***Sup. Table 4 | GeneMatch (2) (Phoenix), USA –Cohort characteristics.*** *Age, MMSE and CDR scores,* *one-way ANOVA followed by Holm-Šídák's multiple comparisons post hoc test. Gender, APOE4 and amyloid status, chi-square test. Post hoc analyses were performed with the non-AD BD clinical group as the reference. *p<0.05, **p<0.01, ***p<0.001. NA, Not Applicable.*

Supplementary Table 5

***
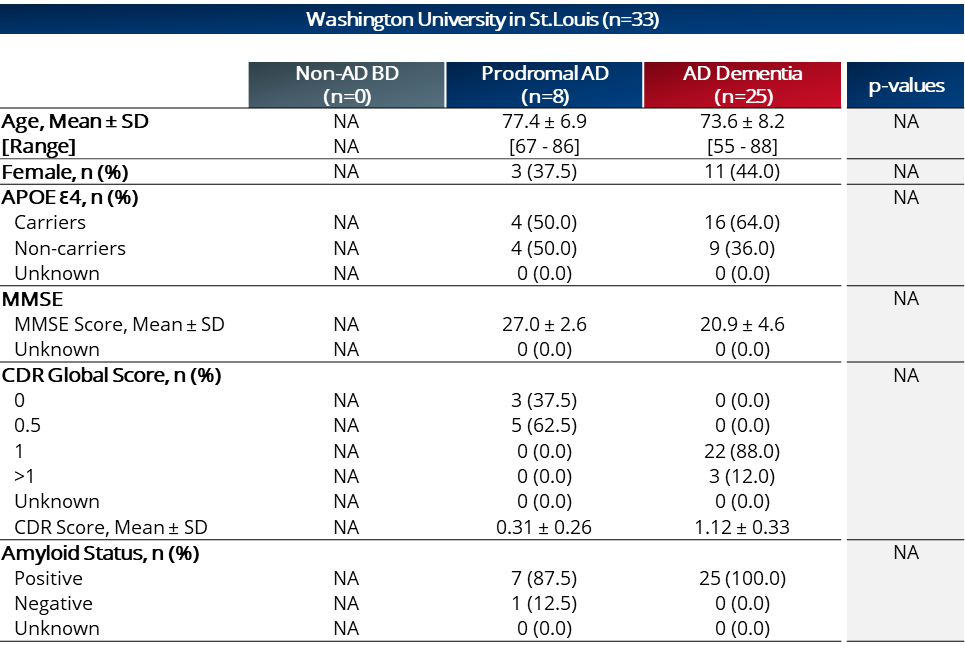
***

***Sup. Table 5 | Washington university (St. Louis), USA – Cohort characteristics.*** *Age, MMSE and CDR scores,* *one-way ANOVA followed by Holm-Šídák’s multiple comparisons post hoc test. Gender, APOE4 and amyloid status, chi-square test. Post hoc analyses were performed with the non-AD BD clinical group as the reference. *p<0.05, **p<0.01, ***p<0.001. NA, Not Applicable.*

Supplementary Table 6


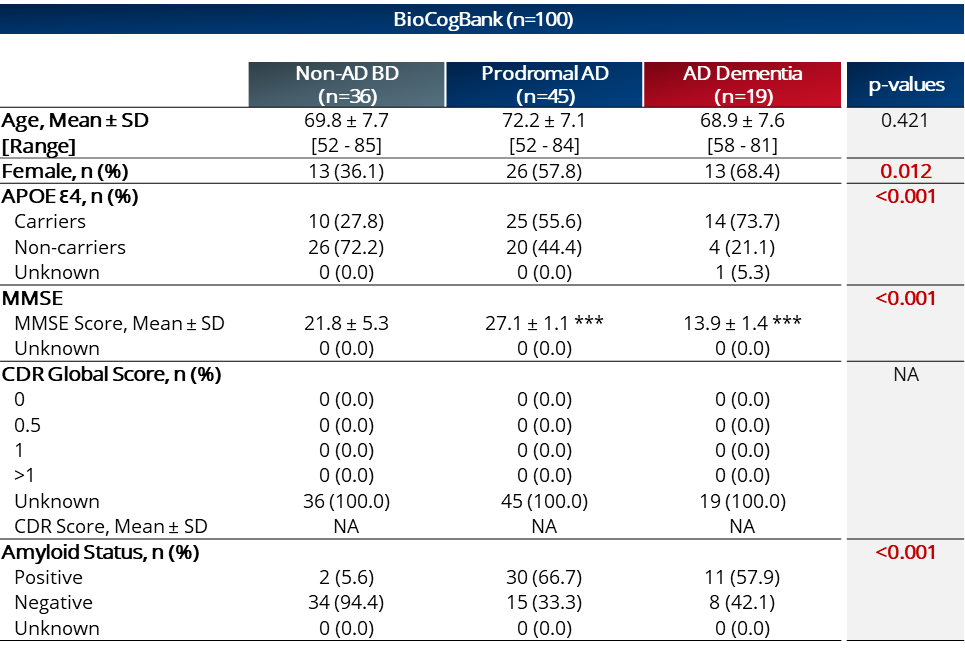


***Sup. Table 6 | BioCogBank (3) (Paris), France – Cohort characteristics.*** *Age, MMSE and CDR scores,* *one-way ANOVA followed by Holm-Šídák's multiple comparisons post hoc test. Gender, APOE4 and amyloid status, chi-square test. Post hoc analyses were performed with the non-AD BD clinical group as the reference. *p<0.05, **p<0.01, ***p<0.001. NA, Not Applicable.*

Supplementary Table 7

***Sup. Table 7 | University of Washington (Seattle), USA – Cohort characteristics.*** *Age, MMSE and CDR scores,* *one-way ANOVA followed by Holm-Šídák's multiple comparisons post hoc test. Gender, APOE4 and amyloid status, chi-square test. Post hoc analyses were performed with the non-AD BD clinical group as the reference. *p<0.05, **p<0.01, ***p<0.001. NA, Not Applicable.*

Supplementary Table 8

***
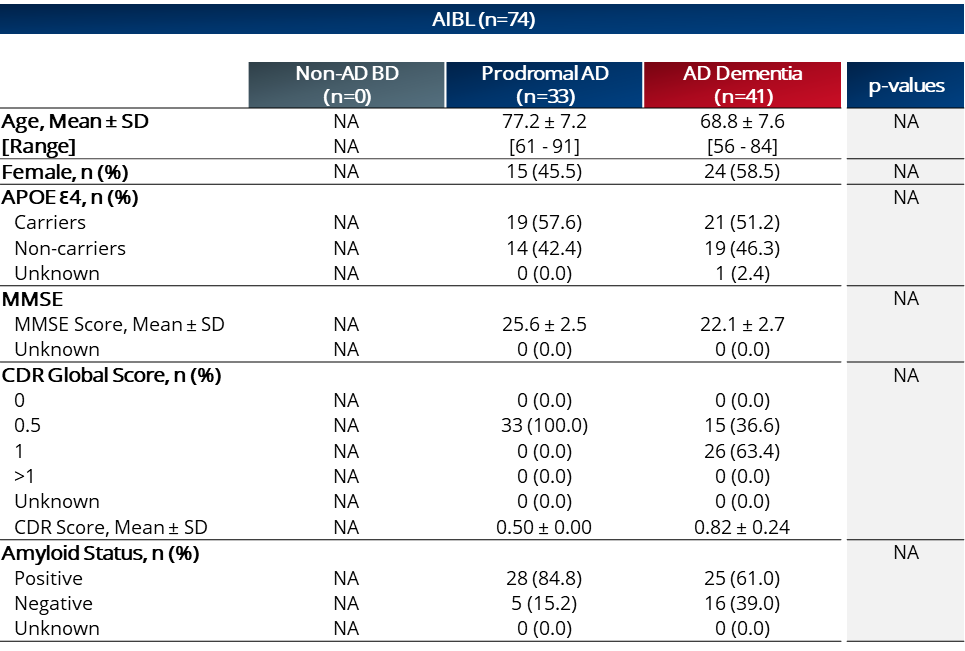
***

***Sup. Table 8 | AIBL (4) (Sydney), Australia – Cohort characteristics.*** *Age, MMSE and CDR scores,* *one-way ANOVA followed by Holm-Šídák's multiple comparisons post hoc test. Gender, APOE4 and amyloid status, chi-square test. Post hoc analyses were performed with the non-AD BD clinical group as the reference. *p<0.05, **p<0.01, ***p<0.001. NA, Not Applicable.*

Supplementary Table 9

***
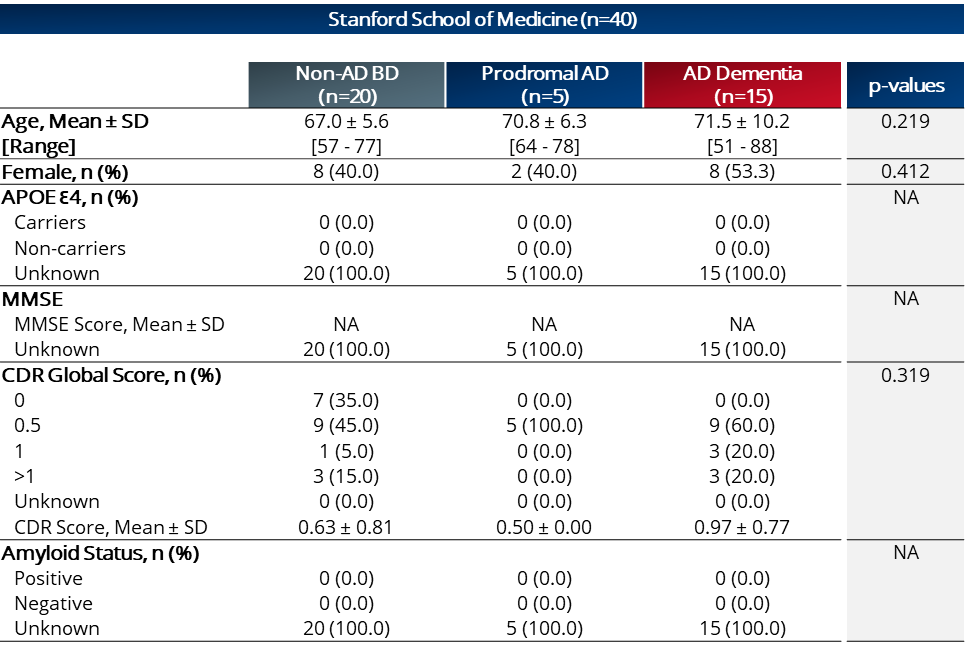
***

***Sup. Table 9 | Stanford School of Medicine (Stanford), USA – Cohort characteristics.*** *Age, MMSE and CDR scores,* *one-way ANOVA followed by Holm-Šídák's multiple comparisons post hoc test. Gender, APOE4 and amyloid status, chi-square test. Post hoc analyses were performed with the non-AD BD clinical group as the reference. *p<0.05, **p<0.01, ***p<0.001. NA, Not Applicable.*

Supplementary Table 10

***
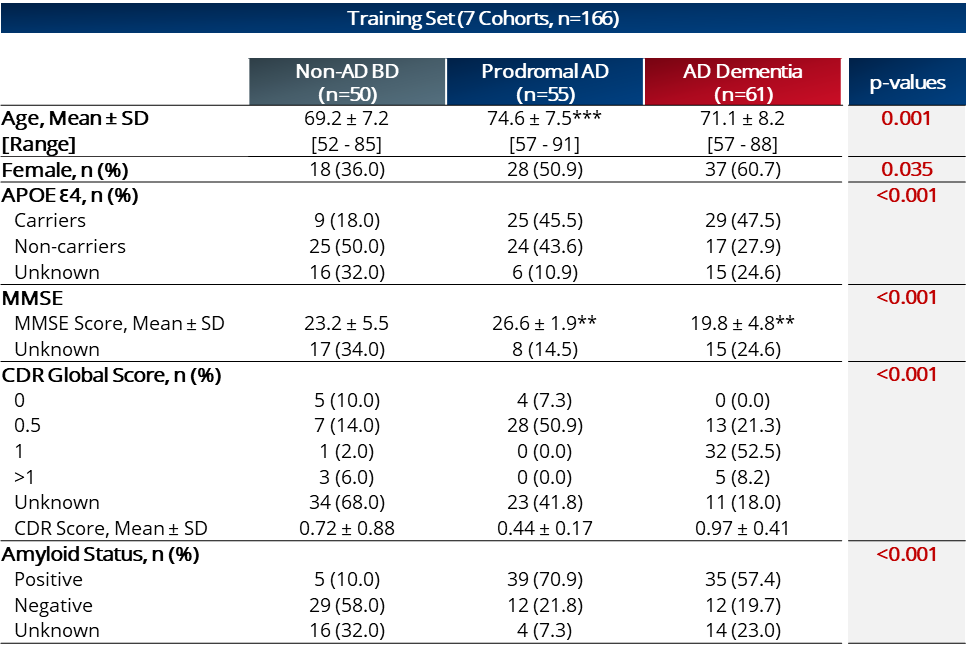
***

***Sup. Table 10 | B-HEALED test cohorts characteristics according to Training, Validation and Test datasets.*** *Age, MMSE and CDR scores,* *one-way ANOVA followed by Holm-Šídák's multiple comparisons post hoc test with the non-AD BD clinical group as the reference. Gender, APOE4 and amyloid status, chi-square test. *p<0.05, **p<0.01, ***p<0.001.*

Supplementary Table 11

***
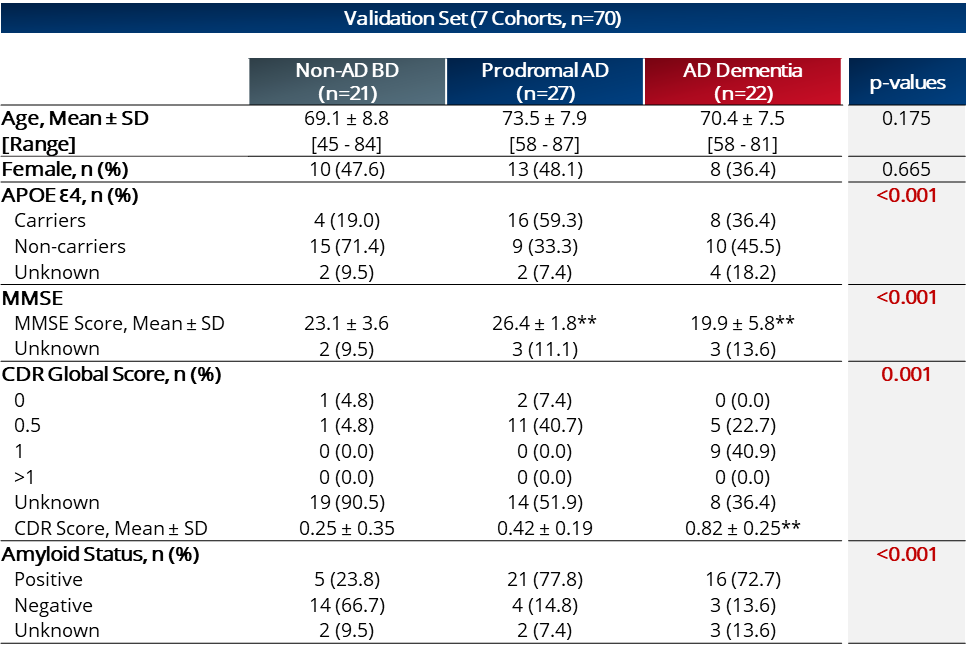
***

***Sup. Table 11 | B-HEALED test cohorts characteristics according to Validation dataset.*** *Age, MMSE and CDR scores,* *one-way ANOVA followed by Holm-Šídák's multiple comparisons post hoc test with the non-AD BD clinical group as the reference. Gender, APOE4 and amyloid status, chi-square test. *p<0.05, **p<0.01, ***p<0.001.*

Supplementary Table 12

***
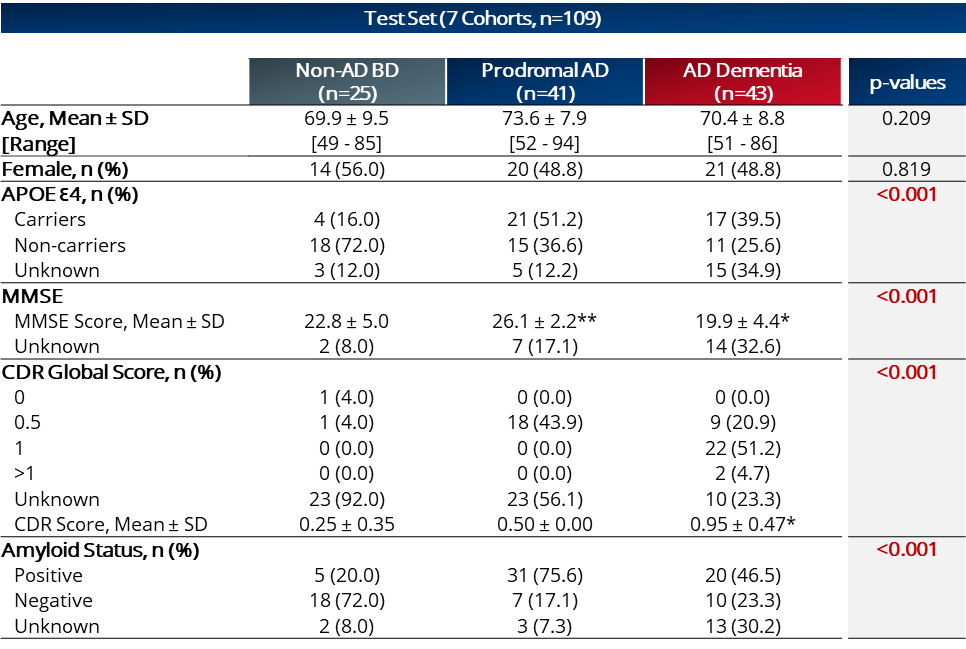
***

***Sup. Table 12 | B-HEALED test cohorts characteristics according to Test dataset.*** *Age, MMSE and CDR scores,* *one-way ANOVA followed by Holm-Šídák's multiple comparisons post hoc test with the non-AD BD clinical group as the reference. Gender, APOE4 and amyloid status, chi-square test. *p<0.05, **p<0.01, ***p<0.001.*

Biomarker biological pathway overview

A total of 19 biomarkers (13 proteins and 6 metabolites) have been quantified by 3 proprietary assay methods based on targeted mass spectrometry. These biomarkers are involved in the following biological pathways similar to those described at the cerebral level:

**Innate immune response (31%)**: Studies have shown that inflammation, a key component of the innate immune system, is involved in the development of AD (5). The activation status of peripheral innate immune cells may represent an early biomarker of the upcoming impact on the brain (6).

**Blood coagulation (16%)**: Studies suggest that the intrinsic/contact activation pathway, which initiates prothrombotic and proinflammatory pathways, is dysregulated in Alzheimer's patients and related animal models (7-11). Aβ42 has been found to form tPA-resistant clots when clotting is triggered directly via thrombin (12-15).

**Lipid metabolism (16%):** The major component of the brain, lipids, have been linked to both healthy brain function and the most common diseases affecting the brain. Demographics, genetics, and lifestyle all affect lipid metabolism, and are significant contributors to lipid disruption as seen in AD (16). In particular, the most common genetic risk factor for AD, APOE4, is involved in lipid transportation and metabolism (17).

**Bioenergetics (11%)**: By combining multiple levels of analysis, from genomic analyses in animal models to in vitro cellular model systems and brain imaging in humans, it has been shown that dysfunctions in glucose metabolism, bioenergetics, and mitochondrial function are linked to the development of Alzheimer's pathology, as demonstrated through a range of experimental paradigms, including postmortem autopsies of human brains **(18-21).**

**Oxidative stress (11%):** Strong evidence suggests that oxidative stress is involved in the pathology of Alzheimer's disease, leading to tau hyperphosphorylation and the formation of neurofibrillary tangles through the inhibition of phosphatase 2A, the activation of glycogen synthase kinase 3β (GSK3β) (22, 23), and the buildup of amyloid-beta due to the ROS-induced suppression of the proteasomal system through impaired mammalian target of rapamycin (mTOR) signaling (24).

**Cell protection (5%)**: Cell protection is an important factor in preventing the development of Alzheimer's disease. By protecting cells from damage caused by oxidative stress and inflammation, the risk of Alzheimer's can be minimized (25).

**Hormonal System (5%):** The effects of testosterone and estradiol in the brain of older individuals remain unclear, however, research suggests that these hormones may provide a variety of neuroprotective benefits (26).

**APP/Aβ metabolism (5%):** Altered amyloid precursor protein (APP) metabolism plays a critical role in the development of AD. APP is a protein that is metabolized and degraded to produce peptide fragments known as amyloid beta (Aβ). Studies have shown that APP metabolism is altered in Alzheimer's disease patients, resulting in increased production and abnormal accumulation of Aβ in the brain. This increase in Aβ levels is considered a key marker of Alzheimer's disease and has been associated with an increased risk of developing the disease. Therefore, APP metabolism is considered as a major risk factor for the development of AD (27).

# Supplementary discussion

We propose an AD continuum in 3 main steps, inspired by the progression of cancers (28): exposition, promotion and progression (**Fig.5c; Supplementary Fig. 7**). The exposition is the result of the increase in the concentration of soluble Aβ42 (oligomers or protofibrils) in the brain but also in the periphery (29). This increase can vary depending on different factors such as age (30), genetic (31) or modifiable factors (32). During this phase, the individual does not yet have AD. The concentration of soluble peptide Aβ42 will increase until it reaches a concentration where the subject's body will respond, as a consequence of the induced toxicity. The promotion phase corresponds to the stage where the Aβ42 concentration has reached the individual threshold for Aβ42-toxicity susceptibility. This threshold can vary from one individual to another which could explain why some individuals develop amyloid plaques without developing symptoms of the disease (high susceptibility threshold) and why some patients develop AD symptoms without amyloid plaques (low susceptibility threshold). This phase will thus be characterized by the activation or deregulation of biological pathways in a manner characteristic of AD: bioenergetics (18-21)**,** cell protection (25), blood coagulation (11), innate immune response (5, 33), sex hormonal system (26), APP/Aβ metabolism (27), oxidative stress (22-24) and lipid metabolism (34). Depending on each individual's intrinsic threshold of susceptibility, the promotion phase will begin earlier or later, depending on genetic or modifiable factors. Finally, the progression phase will encompass the “classical” chain of hallmarks involved in the AD pathology, such as amyloid and tau deposition, neurodegeneration, and progressive cognitive impairment. The biological pathways activated during the promotion phase will be maintained, amplified in intensity but also evolve in parallel with the appearance of hallmarks.

By identifying the molecular pattern specific to the promotion and progression phases, it would be possible to predict with high specificity AD patients regardless of their amyloid status at the time of testing. It is therefore possible to develop tests predicting AD with a specificity not accessible to amyloid tests. However, as for the soluble Aβ42 peptide, it is not possible to estimate these molecular activations at the brain level while the patient is alive (**Fig.5b**). An alternative to measuring these cerebral molecular deregulations would be to measure these alterations in the periphery at the blood level. The soluble Aβ42 peptide can thus be produced at the brain level but will cross the blood-brain barrier mediated by low-density lipoprotein receptor-related peptide 1 (LRP1) (35), drainage from interstitial fluid (ISF) into cerebrospinal fluid (CSF) via perivascular r(36) or glymphatic pathways (37), reabsorption from CSF into the venous blood via arachnoid villi (38) and blood–CSF barrier (39), and possibly via meningeal lymphatic vessels (37, 40) to reach the bloodstream (41). Soluble Aβ42 peptide is also produced by peripheral organs (42). There is a flow, if not an equilibrium, between the soluble amyloid peptide produced at the brain level and that produced at the peripheral level (43) (**Fig.5b**). It is therefore possible to develop tests predicting AD status with a high specificity not accessible to amyloid tests based on peripheral abnormalities (44) as a result of Aβ-related specific AD biological deregulations (**Sup Fig.7**).

By deciphering these peripheral alterations in the blood of AAV-AD rats and then confirming them in a human context, we demonstrated that these alterations involved the same biological pathways as those described at the brain level. This establishes that brain cells and peripheral cells collectively respond to the toxicity induced by soluble amyloid peptides. This peripheral response pattern, therefore, enables the high-specificity prediction that the subject being tested has surpassed the promotion phase. These findings, corroborated by external validation, are thus robust and generalizable. The inclusion of heterogeneity, encompassing aspects such as participants’ geographical origins, gender, associated comorbidities, or non-AD pathology types, and analytical reproducibility, strengthens the applicability of these tests under routine clinical practice.

Identifying populations distinct from those identified by the amyloid deposit-related tests enables their complementary usage. Consequently, the B-HEALED test could serve as an additional instrument for patient inclusion in clinical trials or for informed prescription of developmental or FDA-approved drugs. Potential participants could be pre-screened based on the mechanism of action of the drug to be tested or prescribed. Individuals who test positive could then be evaluated using the B-HEALED test to minimize the risk of misdiagnosis.

# Supplementary References

1. Alcolea D, Clarimon J, Carmona-Iragui M, Illan-Gala I, Morenas-Rodriguez E, Barroeta I, et al. The Sant Pau Initiative on Neurodegeneration (SPIN) cohort: A data set for biomarker discovery and validation in neurodegenerative disorders. Alzheimers Dement (N Y). 2019;5:597-609.

2. Langbaum JB, Karlawish J, Roberts JS, Wood EM, Bradbury A, High N, et al. GeneMatch: A novel recruitment registry using at-home APOE genotyping to enhance referrals to Alzheimer's prevention studies. Alzheimers Dement. 2019;15(4):515-24.

3. Lafirdeen ASM, Cognat E, Sabia S, Hourregue C, Lilamand M, Dugravot A, et al. Biomarker profiles of Alzheimer's disease and dynamic of the association between cerebrospinal fluid levels of beta-amyloid peptide and tau. PLoS One. 2019;14(5):e0217026.

4. Ellis KA, Bush AI, Darby D, De Fazio D, Foster J, Hudson P, et al. The Australian Imaging, Biomarkers and Lifestyle (AIBL) study of aging: methodology and baseline characteristics of 1112 individuals recruited for a longitudinal study of Alzheimer's disease. Int Psychogeriatr. 2009;21(4):672-87.

5. Boutajangout A, Wisniewski T. The innate immune system in Alzheimer's disease. Int J Cell Biol. 2013;2013:576383.

6. Le Page A, Dupuis G, Frost EH, Larbi A, Pawelec G, Witkowski JM, et al. Role of the peripheral innate immune system in the development of Alzheimer's disease. Exp Gerontol. 2018;107:59-66.

7. Bergamaschini L, Donarini C, Gobbo G, Parnetti L, Gallai V. Activation of complement and contact system in Alzheimer's disease. Mech Ageing Dev. 2001;122(16):1971-83.

8. Ashby EL, Love S, Kehoe PG. Assessment of activation of the plasma kallikrein-kinin system in frontal and temporal cortex in Alzheimer's disease and vascular dementia. Neurobiol Aging. 2012;33(7):1345-55.

9. Zamolodchikov D, Chen ZL, Conti BA, Renne T, Strickland S. Activation of the factor XII-driven contact system in Alzheimer's disease patient and mouse model plasma. Proc Natl Acad Sci U S A. 2015;112(13):4068-73.

10. Zamolodchikov D, Renne T, Strickland S. The Alzheimer's disease peptide beta-amyloid promotes thrombin generation through activation of coagulation factor XII. J Thromb Haemost. 2016;14(5):995-1007.

11. Chen ZL, Revenko AS, Singh P, MacLeod AR, Norris EH, Strickland S. Depletion of coagulation factor XII ameliorates brain pathology and cognitive impairment in Alzheimer disease mice. Blood. 2017;129(18):2547-56.

12. Cortes-Canteli M, Paul J, Norris EH, Bronstein R, Ahn HJ, Zamolodchikov D, et al. Fibrinogen and beta-amyloid association alters thrombosis and fibrinolysis: a possible contributing factor to Alzheimer's disease. Neuron. 2010;66(5):695-709.

13. Zamolodchikov D, Strickland S. Abeta delays fibrin clot lysis by altering fibrin structure and attenuating plasminogen binding to fibrin. Blood. 2012;119(14):3342-51.

14. Ahn HJ, Glickman JF, Poon KL, Zamolodchikov D, Jno-Charles OC, Norris EH, et al. A novel Abeta-fibrinogen interaction inhibitor rescues altered thrombosis and cognitive decline in Alzheimer's disease mice. J Exp Med. 2014;211(6):1049-62.

15. Zamolodchikov D, Strickland S. A possible new role for Abeta in vascular and inflammatory dysfunction in Alzheimer's disease. Thromb Res. 2016;141 Suppl 2:S59-61.

16. Chew H, Solomon VA, Fonteh AN. Involvement of Lipids in Alzheimer's Disease Pathology and Potential Therapies. Front Physiol. 2020;11:598.

17. Strittmatter WJ, Roses AD. Apolipoprotein E and Alzheimer's disease. Annu Rev Neurosci. 1996;19:53-77.

18. Gibson GE, Shi Q. A mitocentric view of Alzheimer's disease suggests multi-faceted treatments. J Alzheimers Dis. 2010;20 Suppl 2(0 2):S591-607.

19. Yao J, Irwin RW, Zhao L, Nilsen J, Hamilton RT, Brinton RD. Mitochondrial bioenergetic deficit precedes Alzheimer's pathology in female mouse model of Alzheimer's disease. Proc Natl Acad Sci U S A. 2009;106(34):14670-5.

20. Nicholson RM, Kusne Y, Nowak LA, LaFerla FM, Reiman EM, Valla J. Regional cerebral glucose uptake in the 3xTG model of Alzheimer's disease highlights common regional vulnerability across AD mouse models. Brain Res. 2010;1347:179-85.

21. Hauptmann S, Scherping I, Drose S, Brandt U, Schulz KL, Jendrach M, et al. Mitochondrial dysfunction: an early event in Alzheimer pathology accumulates with age in AD transgenic mice. Neurobiol Aging. 2009;30(10):1574-86.

22. Elgenaidi IS, Spiers JP. Regulation of the phosphoprotein phosphatase 2A system and its modulation during oxidative stress: A potential therapeutic target? Pharmacol Ther. 2019;198:68-89.

23. Toral-Rios D, Pichardo-Rojas PS, Alonso-Vanegas M, Campos-Pena V. GSK3beta and Tau Protein in Alzheimer's Disease and Epilepsy. Front Cell Neurosci. 2020;14:19.

24. Perluigi M, Di Domenico F, Barone E, Butterfield DA. mTOR in Alzheimer disease and its earlier stages: Links to oxidative damage in the progression of this dementing disorder. Free Radic Biol Med. 2021;169:382-96.

25. Morris MC, Tangney CC, Wang Y, Sacks FM, Bennett DA, Aggarwal NT. MIND diet associated with reduced incidence of Alzheimer's disease. Alzheimers Dement. 2015;11(9):1007-14.

26. Veiga S, Melcangi RC, Doncarlos LL, Garcia-Segura LM, Azcoitia I. Sex hormones and brain aging. Exp Gerontol. 2004;39(11-12):1623-31.

27. Zhang YW, Thompson R, Zhang H, Xu H. APP processing in Alzheimer's disease. Mol Brain. 2011;4:3.

28. Pitot HC. The molecular biology of carcinogenesis. Cancer. 1993;72(3 Suppl):962-70.

29. Zou L, Yang R, Zhang P, Dai Y. The enhancement of amyloid precursor protein and beta-site amyloid cleavage enzyme 1 interaction: amyloid-beta production with aging. Int J Mol Med. 2010;25(3):401-7.

30. Guerreiro R, Bras J. The age factor in Alzheimer's disease. Genome Med. 2015;7:106.

31. Chouraki V, Seshadri S. Genetics of Alzheimer's disease. Adv Genet. 2014;87:245-94.

32. Edwards Iii GA, Gamez N, Escobedo G, Jr., Calderon O, Moreno-Gonzalez I. Modifiable Risk Factors for Alzheimer's Disease. Front Aging Neurosci. 2019;11:146.

33. Tenner AJ. Complement in Alzheimer's disease: opportunities for modulating protective and pathogenic events. Neurobiol Aging. 2001;22(6):849-61.

34. Song F, Poljak A, Crawford J, Kochan NA, Wen W, Cameron B, et al. Plasma apolipoprotein levels are associated with cognitive status and decline in a community cohort of older individuals. PLoS One. 2012;7(6):e34078.

35. Shibata M, Yamada S, Kumar SR, Calero M, Bading J, Frangione B, et al. Clearance of Alzheimer's amyloid-ss(1-40) peptide from brain by LDL receptor-related protein-1 at the blood-brain barrier. J Clin Invest. 2000;106(12):1489-99.

36. Preston SD, Steart PV, Wilkinson A, Nicoll JA, Weller RO. Capillary and arterial cerebral amyloid angiopathy in Alzheimer's disease: defining the perivascular route for the elimination of amyloid beta from the human brain. Neuropathol Appl Neurobiol. 2003;29(2):106-17.

37. Iliff JJ, Wang M, Liao Y, Plogg BA, Peng W, Gundersen GA, et al. A paravascular pathway facilitates CSF flow through the brain parenchyma and the clearance of interstitial solutes, including amyloid beta. Sci Transl Med. 2012;4(147):147ra11.

38. Silverberg GD, Mayo M, Saul T, Rubenstein E, McGuire D. Alzheimer's disease, normal-pressure hydrocephalus, and senescent changes in CSF circulatory physiology: a hypothesis. Lancet Neurol. 2003;2(8):506-11.

39. Pascale CL, Miller MC, Chiu C, Boylan M, Caralopoulos IN, Gonzalez L, et al. Amyloid-beta transporter expression at the blood-CSF barrier is age-dependent. Fluids Barriers CNS. 2011;8:21.

40. Louveau A, Smirnov I, Keyes TJ, Eccles JD, Rouhani SJ, Peske JD, et al. Structural and functional features of central nervous system lymphatic vessels. Nature. 2015;523(7560):337-41.

41. Xiang Y, Bu XL, Liu YH, Zhu C, Shen LL, Jiao SS, et al. Physiological amyloid-beta clearance in the periphery and its therapeutic potential for Alzheimer's disease. Acta Neuropathol. 2015;130(4):487-99.

42. Citron M, Vigo-Pelfrey C, Teplow DB, Miller C, Schenk D, Johnston J, et al. Excessive production of amyloid beta-protein by peripheral cells of symptomatic and presymptomatic patients carrying the Swedish familial Alzheimer disease mutation. Proc Natl Acad Sci U S A. 1994;91(25):11993-7.

43. Ullah R, Park TJ, Huang X, Kim MO. Abnormal amyloid beta metabolism in systemic abnormalities and Alzheimer's pathology: Insights and therapeutic approaches from periphery. Ageing Res Rev. 2021;71:101451.

44. Trushina E. Alzheimer's disease mechanisms in peripheral cells: Promises and challenges. Alzheimers Dement (N Y). 2019;5:652-60.
